# Mechanistic study of the hsa_circ_0074158 binding EIF4A3 impairing sepsis-induced endothelial barrier

**DOI:** 10.3389/fimmu.2025.1621095

**Published:** 2025-09-22

**Authors:** Haiyan Liao, Yiming Li, Li Zhang, Zhiyong Peng, Zhaohui Zhang

**Affiliations:** ^1^ Department of Critical Care Medicine, Zhongnan Hospital of Wuhan University, Wuhan, Hubei, China; ^2^ Departments of Critical Care Medicine, Yichang Central People’s Hospital, Yichang, Hubei, China; ^3^ The First College of Clinical Medical Science, China Three Gorges University, Yichang, Hubei, China

**Keywords:** sepsis, hsa_circ_0074158, EIF4A3, RNA-binding protein, endothelial barrier function

## Abstract

**Background:**

Sepsis remains a major clinical challenge, characterized by high rates of morbidity, mortality, and healthcare burden. Its complex pathogenesis involves multiple factors, with damage to the vascular endothelial barrier being a key component. Currently, there are no widely accepted biomarkers that demonstrate high sensitivity and specificity for sepsis, and treatment mainly focuses on supportive care without specific therapeutic targets. Recent research suggests that circRNA has potential as both a biomarker and a therapeutic target. In previous studies, the harmful role of hsa_circ_0074158 in worsening sepsis-induced endothelial barrier dysfunction was demonstrated through both *in vitro* and *in vivo* models, highlighting its potential as a biomarker and therapeutic target for sepsis.

**Methods:**

This study examined how hsa_circ_0074158 affects endothelial barrier function in sepsis. The role of hsa_circ_0074158 (circ_Ctnna1 in mice) in sepsis-related endothelial barrier dysfunction was studied using both *in vitro* and *in vivo* models. RNA pull-down, RNA immunoprecipitation (RIP), and actinomycin D experiments were used to show that circ_0074158 impacts endothelial barrier function in sepsis by reducing the stability of the host gene CTNNA1 (mRNA) after binding to EIF4A3.

**Results:**

In both LPS-treated human umbilical vein endothelial cells (HUVECs) and cecal ligation and puncture (CLP) murine models, the overexpression of hsa_circ_0074158 (the mouse homolog of hsa_circ_0074158 is named circ_Ctnna1) significantly decreased CTNNA1 mRNA stability and increased endothelial hyperpermeability, while its knockdown restored barrier integrity. Mechanistically, RNA pull-down and RNA RIP assays demonstrated that hsa_circ_0074158 directly binds to the RNA-binding protein (RBP) EIF4A3, which decreases the stability of CTNNA1 (mRNA) and the production of α-catenin, subsequently impairing endothelial barrier function in sepsis. Rescue experiments showed that dual targeting of hsa_circ_0074158 and EIF4A3 restored endothelial barrier function. The dysregulation of the hsa_circ_0074158/EIF4A3 axis exacerbates sepsis-induced endothelial barrier dysfunction by destabilizing CTNNA1 mRNA, posing a critical medical challenge due to its complex pathophysiology.

**Conclusions:**

This study provides new insights into the molecular mechanisms of sepsis and suggests potential therapeutic targets for its treatment. Further research is needed to explore the clinical application of these findings.

## 1 Introduction

Sepsis, characterized by its high incidence and mortality rates, has been a significant concern due to its substantial healthcare burden (1). The “Surviving Sepsis Campaign” aims to reduce sepsis and septic shock incidence and mortality rates; however, despite over two decades of effort, the results remain disappointing (2). Approximately 48.9 million cases of sepsis occur globally each year, accounting for 19.7% of all deaths. Moreover, healthcare costs associated with sepsis continue to rise annually (3–5). The pathogenesis of sepsis is complex and primarily involves damage to the vascular endothelial barrier, increased vascular permeability, inflammatory response, immune response, and coagulation dysfunction, with damage to the vascular endothelial barrier being particularly critical (6). Reports indicate that circular RNAs (circRNAs) play a role in the pathogenesis of sepsis in various ways and are correlated with disease severity, risk, and patient prognosis (7). Our previous research suggests that circRNA may regulate endothelial barrier function in sepsis (8).

Sepsis currently lacks biomarkers with high sensitivity and specificity that are universally accepted. Treatment mainly focuses on supportive care, with no specific treatment options or effective therapeutic targets available. With the development of bioinformatics, some studies have demonstrated the potential of circRNA as both a biomarker and a therapeutic target, suggesting that circRNA could offer new prospects for diagnosing and treating clinical diseases (7, 9–12). Due to their unique circular structure and high stability, specificity, and conservation, circRNAs are considered highly promising clinical biomarkers and have become a focal point of current research. Studies have found that circRNA can regulate gene expression through multiple pathways (11, 13, 14), such as controlling transcription and splicing, acting as miRNA sponges, and modulating host gene expression.

Furthermore, abnormal circRNA regulation has been associated with diseases like cancer and sepsis (11, 15, 16). To investigate the correlation between circRNAs and sepsis, we conducted RNA-seq in sepsis patients and identified a significantly differentially expressed circRNA, hsa_circ_0074158 (8). Circular RNA hsa_circ_0074158, located on human chromosome 5, is a non-coding RNA with a closed circular structure, derived from the exons 3 and 4 of its host gene CTNNA1, encoding α-catenin. α-catenin is a crucial protein for maintaining vascular endothelial barrier function, essential for the integrity of endothelial structure, cell-cell junctions, and glycocalyx. Tight junctions, adherens junctions, and desmosomes maintain intercellular junctions. Adherens junctions, centered on vascular endothelial cadherin (VE-cadherin), form an important structure of the endothelial barrier (17). VE-cadherin combines with catenins such as α-catenin, affecting the integrity and stability of the vascular endothelium (18). The glycocalyx, which covers the surface of endothelial cells, maintains vascular homeostasis, with its main components being syndecan-1, heparan sulfate, and hyaluronan, which are considered biomarkers of glycocalyx degradation (19–21). In sepsis, damage to the vascular endothelial barrier increases cellular permeability, leading to impaired fluid exchange between the vasculature and interstitium, resulting in edema, reduced tissue perfusion, organ damage, and potentially life-threatening organ failure.

The current understanding of sepsis-induced endothelial barrier dysfunction has been significantly advanced by studies elucidating the role of specific circRNAs, such as hsa_circ_0074158 (8). Preliminary investigations in cellular models have demonstrated that hsa_circ_0074158 contributes to the impairment of the endothelial barrier in sepsis, and clinical studies confirmed its high expression in sepsis patients, which is correlated with the patient’s underlying health status, disease severity, and prognosis. However, the molecular mechanism remains unclear. In this study, we aimed to elucidate the molecular mechanism of hsa_circ_0074158 in sepsis. We used various research methods to investigate the expression and function of hsa_circ_0074158 in sepsis. Our results showed that hsa_circ_0074158 regulates its host gene CTNNA1 (mRNA) to affect the endothelial barrier function in sepsis.

Additionally, hsa_circ_0074158 binds to the RNA-binding protein EIF4A3 to form an RNA-binding protein (RBP) complex, which reduces the stability of CTNNA1 (mRNA) and the generation of α-catenin, thereby damaging the endothelial barrier function in sepsis. Our findings provide new insights into the molecular mechanism of sepsis and suggest that hsa_circ_0074158 may be a potential biomarker and therapeutic target for sepsis. Further studies are needed to validate these findings and explore the clinical application of hsa_circ_0074158 in sepsis management.

## 2 Materials and methods

### 2.1 Cell culture and cell treatment

The human umbilical vein endothelial cells (HUVECs) were available from the Institute of Immunology, Tsinghua University (Beijing, China) and were cultured in endothelial cell medium (ECM; Keycell, Wuhan, China) supplemented with 10% fetal bovine serum (FBS; TianHang, China) and 1% penicillin/streptomycin (Corning, China). Jikai Gene Technology Co. Ltd. (Beijing, China) constructed the lentivirus required for transfection. After transfection, the LPS-induced sepsis group was treated with 1000 ng/ml LPS for 12 h. LPS was purchased from Sigma-Aldrich Co. Ltd (Cat NO. L4516, St Louis, USA).

### 2.2 Animal study

All procedures with animals were approved by the Institutional Animal Care and Use Committee (IACUC) of the Laboratory Animal Research Center, Tsinghua University. 8-week-old male C57BL/6J mice weighing 22 ± 2g, housed in SPF-grade facilities. All mice were administered adeno-associated virus (AAV) by tail-vein injection. AAVs (10×10^11^ transducing units in 200 μl of phosphate buffer saline) were purchased from Jikai Gene Technology Co. Ltd. (Beijing, China). After 8 weeks, the efficiency of overexpression was confirmed via quantitative real-time polymerase chain reaction (RT-qPCR). A cecal ligation and puncture (CLP) surgical operation was performed to establish a sepsis model in mice.

### 2.3 RT-qPCR

Total RNA was extracted with Trizol Reagent (Cat NO. 15596-026, Ambion, Texas, USA) with or without RNase R (20U, 37°C, 30 min; Epicentre Technologies, Madison, WI). Specific convergent primers spanning the circRNA back-splice junction site were designed using the circPrimer (version 2.0). Other primers were designed similarly by Oligo 7 (version 7.37) software. The cDNA was synthesized with the PrimeScript™ RT reagent kit (TOLOBIO, Jiangsu, China). RT-qPCR was carried out in triplicate (biological & technical) with SYBR Green PCR Master Mix (ABI, NY, USA), analyzed by the 2^-ΔΔCt^ method normalized to GAPDH.

### 2.4 Western blot

Total protein was extracted from HUVECs/tissues using radioimmunoprecipitation assay buffer (Wuhan Servicebio Technology Co. Ltd. Hubei, China). Proteins were separated by SDS-PAGE and transferred to activated PVDF membranes (Millipore, MA, USA). After blocking with 5% skimmed milk at room temperature for 2 h, membranes were incubated overnight at 4°C with primary antibodies: VE-cadherin (1:200, Santa Cruz, CA, USA), EIF4A3 (1:2000, Keycell, Wuhan, China), α-catenin (1:2000, Keycell, Wuhan, China) as loading control. Horseradish peroxidase (HRP)-labeled secondary antibodies (Beyotime Biotechnology, Shanghai, China; Keycell, Wuhan, China) were incubated for 1 h followed by chemiluminescence detection. A GAPDH antibody (1:1000, Goodhere Biotechnology, Hangzhou, China) was used as a protein loading control.

### 2.5 Immunofluorescence staining

HUVECs were cultured as monolayers and fixed with 4% paraformaldehyde for 30 min. After blocking with 5% normal goat serum for 1 h, cells were incubated with appropriately diluted primary antibodies (according to manufacturer’s protocol) at 4°C overnight. Following cold PBS washing, Cy3-labeled secondary antibody was applied under dark conditions. Nuclei were counterstained with DAPI (Beyotime Biotechnology, Shanghai, China), and protein expression was analyzed using confocal microscopy (Lycra, Germany).

### 2.6 Hematoxylin-Eosin staining

Collected the right lower lung tissue from mice, fixed it with 4% paraformaldehyde, removed the fixative after 48 h, washed it, and immersed the right lower lung tissue in alcohol for dehydration. Then, the lung tissue in a paraffin embedding machine for embedding, followed by sectioning and baking, dewaxing in xylene, alcohol cleaning, hematoxylin staining, fading in differentiation solution, PBS washing, eosin staining, and finally alcohol dehydration, xylene transparency, and gum sealing. Scanned the sections using a digital slide scanner and analyzed the results (Lycra, Germany).

### 2.7 The Lung injury score

To evaluate lung injury in septic mice, the right lower lung tissue was stained with HE. The slides were reviewed by two professionals in a double-blind manner, using the Smith scoring system. For each parameter, 20 high-power fields (400×) were randomly selected and scored. Additionally, 5 fields were graded based on the Smith scoring results.

### 2.8 Evans blue staining

Mice received tail vein injection of 2% Evans blue (Shanghai hui Biotechnology, Shanghai, China). After 1 h anesthesia, systemic perfusion with 10 mL PBS was performed via left ventricle-right atrial route. Following 15-min perfusion, lung tissues were excised, weighed, and homogenized after PBS washing. Tissue homogenates (100 mg wet tissue/3 mL formamide) were incubated at 37°C overnight. Supernatants were collected by centrifugation and Evans blue content was quantified spectrophotometrically (Lycra, Germany) at 620 nm.

### 2.9 Wet-dry weight ratio

Taken the left lung of the mice, sucked up the surface liquid of the lung with absorbent paper, and accurately weighed and recorded the liquid as wet weight. Baked at 60°C for 72 h, weighed the dry weight, continued baking until the constant weight was reached (this was the final dry weight), and calculated the ratio of lung wet to dry weight.

### 2.10 RNA immunoprecipitation

RNA-Binding Protein Immunoprecipitation Kit (Cat NO. 17-700, Millipore, MA, USA) was available for RIP assay. HUVECs (1 × 10^7^) were lysed with RIP lysis buffer and then incubated with RIP buffer adding the magnetic beads and specific antibodies to EIF4A3 (Cat NO. 17504-1-AP, Keycell, Wuhan, China) and IgG (Cat NO. 3420, Cell Signaling Technology, MA, USA). RT-qPCR was carried out to detect co-precipitated RNAs. Three independent biological and technical duplications were conducted.

### 2.11 Fluorescence *in situ* hybridization

Fluorescein-labeled hsa_circ_0074158 probes (Robobio, Guangzhou, China) were used for FISH in fixed HUVECs (4% paraformaldehyde, Servicebio, Wuhan, China). After overnight probe hybridization, nuclei were counterstained with DAPI (Beyotime Biotechnology, Shanghai, China). Imaging was performed using an Olympus BX53 microscope and confocal microscopy (Olympus BX53 biological microscope), with signal detection via Fluorescent *In Situ* Hybridization Kit (Servicebio, Wuhan, China).

### 2.12 RNA pull-down assay and mass spectrometry

Biotin-labeled probes (Robobio, Guangzhou, China) and an RNA-Binding Protein Immunoprecipitation Kit (Millipore, MA, USA) were used for RNA pull-down in HUVECs (1 × 10^7^). Lysates were incubated with Streptavidin Magnetic Beads (Thermo Fisher Scientific, MA, USA) at room temperature (1 h). RNA and proteins were extracted, and RNA-binding proteins were analyzed via mass spectrometry (Wuhan Institute of Biotechnology, Wuhan, China).

### 2.13 Actinomycin D assay

HUVECs were treated with LPS (1000 ng/ml) for 12 h. And then culture the HUVECs in an endothelial cell medium containing 500 ng/mL actinomycin D (Cat NO. 50-76-0, Merck, Darmstadt, Germany) The HUVECs are collected for RT-qPCR at different times (0 h, 4 h, 8 h, 12 h, 24 h).

### 2.14 Statistical analysis

Data were analyzed using SPSS 26.0 and expressed as mean ± SD. Group comparisons were performed via one-way ANOVA (multiple groups) or Student’s t-test (two groups), categorical variables by chi-square/Fisher’s exact test, and survival outcomes by log-rank test/Cox regression. Statistical significance was defined as *P* < 0.05. Graphs were generated with GraphPad Prism 8.0.2.

## 3 Results

### 3.1 Downregulation of hsa_circ_0074158 could alleviate endothelial barrier dysfunction in sepsis-induced mice.

**Figure 1:**
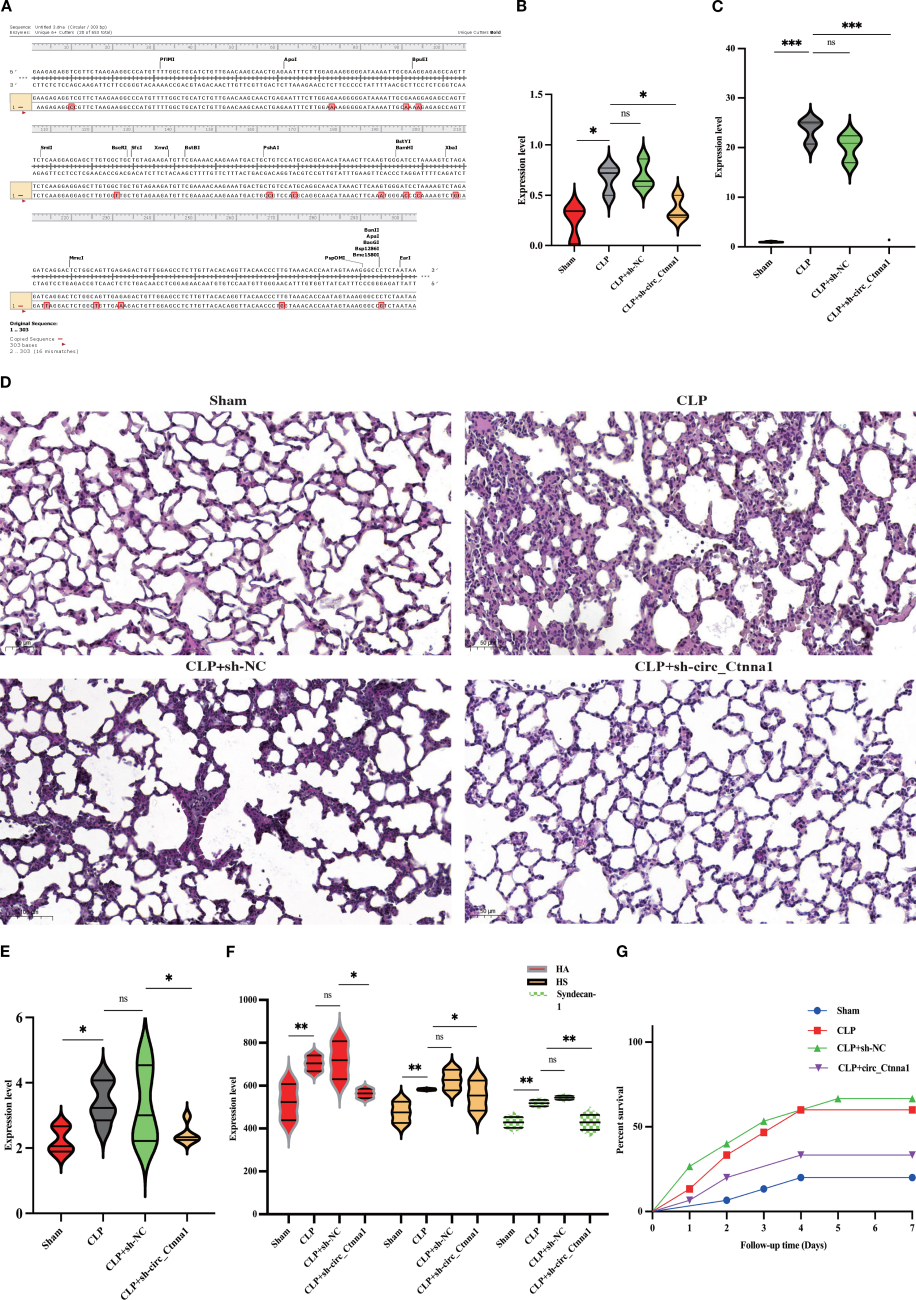
Downregulation of hsa_circ_0074158 alleviated endothelial barrier dysfunction in sepsis-induced mice. **(A)** The circBank showed that hsa_circ_0074158 and mmu_circ_0000860 are homologous, we designate mmu_circ_0000860 as circ_Ctnna1 for further study. **(B)** Lung injury score of mice. **(C)** Lung Evans blue staining. **(D)** HE staining of the lung (200 ×). **(E)** Wet-dry weight ratio of mice lung tissue. **(F)** Serum glycocalyx degradation of mice was measured by ELISA. **(G)** Survival analysis of septic mice. ^***^ *p*<0.001, ^**^ *p*<0.01, ^*^ *p*<0.05.

The current understanding of sepsis pathophysiology highlights the crucial role of endothelial barrier dysfunction in the disease’s progression. Traditional approaches have mainly focused on targeting inflammatory mediators and activating immune cells, yet the underlying mechanisms contributing to endothelial barrier disruption are still not fully understood. Recent studies have implicated circRNAs as key regulators in various pathological processes, including sepsis. Among these, hsa_circ_0074158 has been identified as a potential modulator of endothelial cell function, especially in maintaining barrier integrity. Findings from this investigation demonstrate that the downregulation of hsa_circ_0074158 expression significantly mitigates endothelial barrier dysfunction in a murine model of sepsis. To identify circRNAs associated with CTNNA1, we searched the circBase database (http://www.circbase.org/), revealing 20 human circRNAs and 2 mouse homolog circRNAs associated with CTNNA1 (**Supplementary Table 1**). Upon searching circBank (http://www.circbank.cn/), we discovered that hsa_circ_0074158 and mmu_circ_0000860 are homologous (**Figure 1A**), leading us to designate mmu_circ_0000860 as circ_Ctnna1 for further study. AAV was employed to downregulate circ_Ctnna1 in mice. We induced sepsis in mice via the CLP method to explore the impact of hsa_circ_0074158 on the endothelial barrier in sepsis. Measurements including lung injury score, Evans blue staining, HE staining, wet-dry weight ratio, and serum glycocalyx degradation products in septic mice indicated that downregulation of circ_Ctnna1 could reduce lung injury, alleviate endothelial hyperpermeability, decrease serum glycocalyx degradation (**Figures 1B-F**), and lower the 7-day mortality rate in mice (**Figure 1G**). These results underscore the significance of hsa_circ_0074158 in the pathogenesis of sepsis-induced endothelial injury and suggest that targeting hsa_circ_0074158 may offer a novel therapeutic strategy for preserving endothelial barrier function in sepsis.

### 3.2 The RBPs interacting with hsa_circ_0074158 may be involved in the development of sepsis

**Figure 2:**
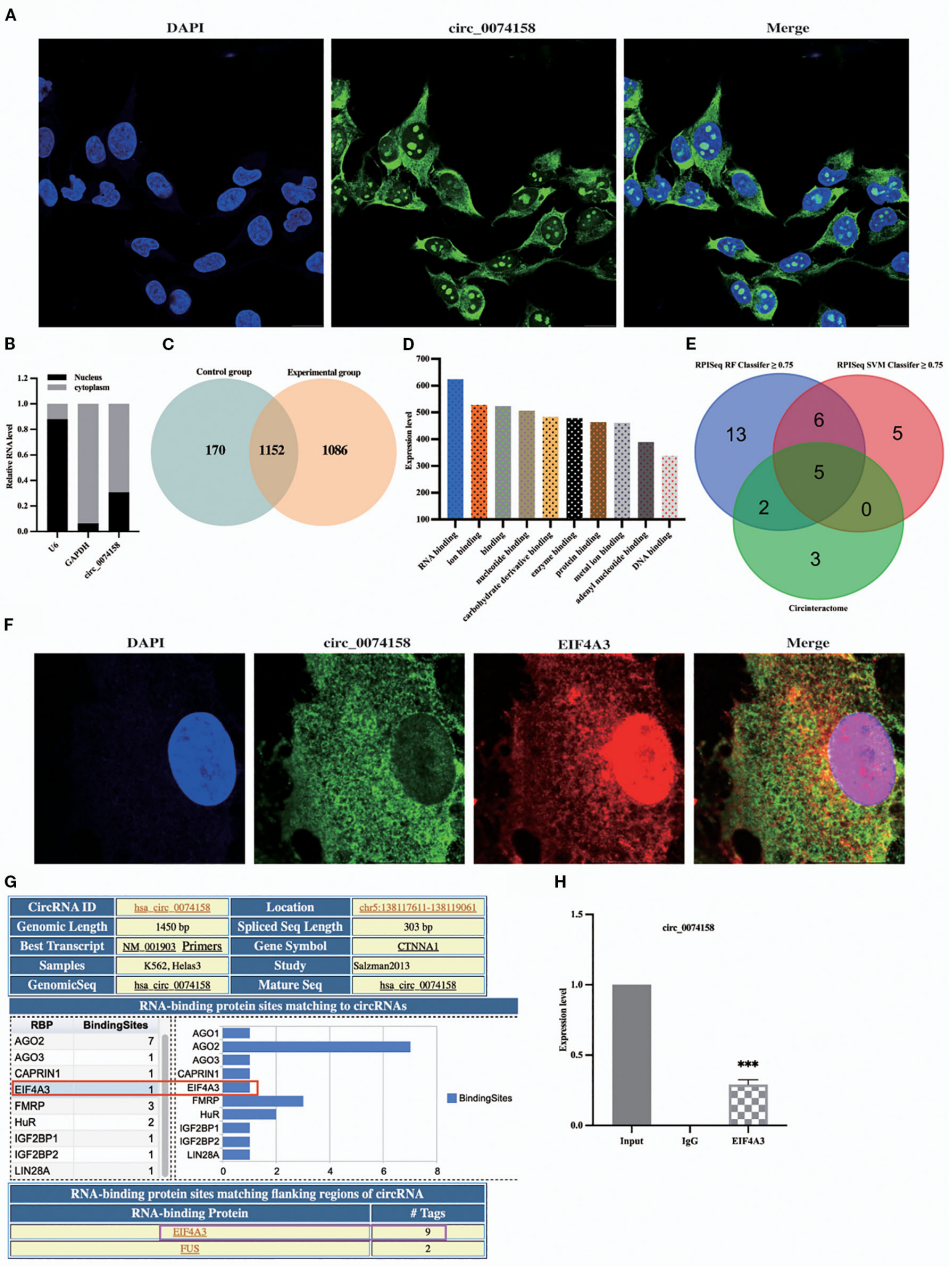
hsa_circ_0074158 interacts with RBP. **(A, B)** FISH and nucleocytoplasmic separation experiments. **(C)** Venn diagram of proteins between different samples. **(D)** The results of mass spectrometry showed that hsa_circ_0074158 could combine with RBP. **(E)** RBPs bound to hsa_circ_0074158 predicted by the database. Bar=20μm. **(F)** hsa_circ_0074158 co-localized with EIF4A3. **(G)** hsa_circ_0074158 and EIF4A3 protein binding region. **(H)** RIP assay showed that hsa_circ_0074158 is bound to EIF4A3. ^***^ *p*<0.001, ^**^ *p*<0.01, ^*^ *p*<0.05.

To further explore the mechanism of the effect of hsa_circ_0074158 on endothelial barrier function in sepsis, cellular experiments were conducted. We found that hsa_circ_0074158 was distributed in both the nucleus and the cytoplasm, suggesting that hsa_circ_0074158 may have a biological function in both the nucleus and the cytoplasm (**Figures 2A, B**). To investigate the pathogenesis of hsa_circ_0074158 in sepsis, we performed RNA pull-down experiments and mass spectrometry. By analyzing the mass spectrometry, the qualitative or quantitative information of the proteins was selected, and the experimental group and the control group proteins were identified with 1086 hsa_circ_0074158 specific binding proteins (**Figure 2C**). The analytical protein results showed that hsa_circ_0074158 bound to RBP more highly than the other proteins (**Figure 2D**). RBP is a class of proteins that can associate with RNA molecules and accompany RNA to regulate metabolic processes. The interaction between circRNA and RBP can affect the mRNA stability of host genes (11, 14, 22, 23), thus affecting tumor progression. However, the effect of hsa_circ_0074158 combined with RBP on the endothelial barrier function in sepsis state was not reported. Meanwhile, we predicted RBP combined with hsa_circ_0074158 by CircInteractome, RPIseq, and RPIseq SVM databases and drew a Wayn diagram, which showed a high probability of hsa_circ_0074158 combined with AGO 1, AGO 2, AGO 3, EIF4A3 and CAPRIN1 (**Figure 2E**). Therefore, we speculated that hsa_circ_0074158 might bind to RBP, where the above five RBP are more likely. We have found that hsa_circ_0074158 and EIF4A3 colocalized (**Figure 2F**). The Circular RNA Interactome database (https://circinteractome.nia.nih.gov/) shows EIF4A3 binding to the gene sequence and flanking region of hsa_circ_0074158, suggesting that EIF4A3 may not only interact with hsa_circ_0074158 but also affect the generation of hsa_circ_0074158 (**Figure 2G**; **Supplementary Figure 1**). More importantly, we have found that hsa_circ_0074158 forms a complex with EIF4A3 (**Figure 2H**). Thus, we speculated that EIF4A3 may be associated with sepsis endothelial barrier function and participate in the development of sepsis.

### 3.3 hsa_circ_0074158 combined with EIF4A3 is involved in the regulation of endothelial barrier function in sepsis

**Figure 3:**
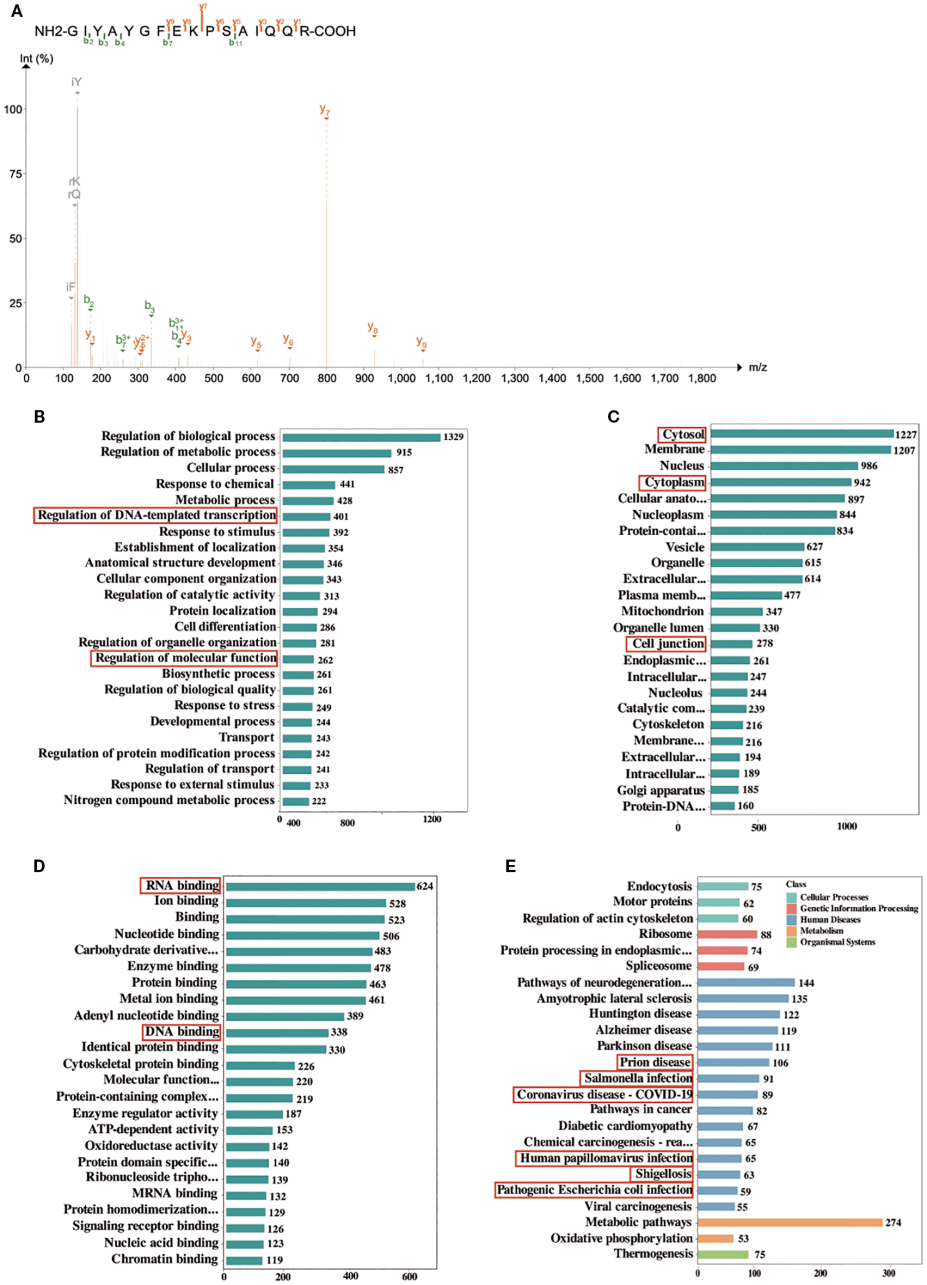
GO and KEGG enrichment analysis of proteins identified by mass spectrometry. **(A)** Mass spectrum of EIF4A3. **(B, C)**, and **(D)** GO enrichment analysis. Each bar shows a type of GO entry, each row represents a GO entry, the abscissa represents the number of proteins identified in this entry, and only the top 24 GO entries are shown in the figure. **(E)** KEGG pathways. The bar graph represents the KEGG pathways annotated by the proteins, the abscissa represents the number of proteins identified in this entry, the ordinate represents the KEGG pathways, and different colors represent different classifications of the pathway. Only the top 24 KEGG pathways are shown in the figure.

**Figure 4:**
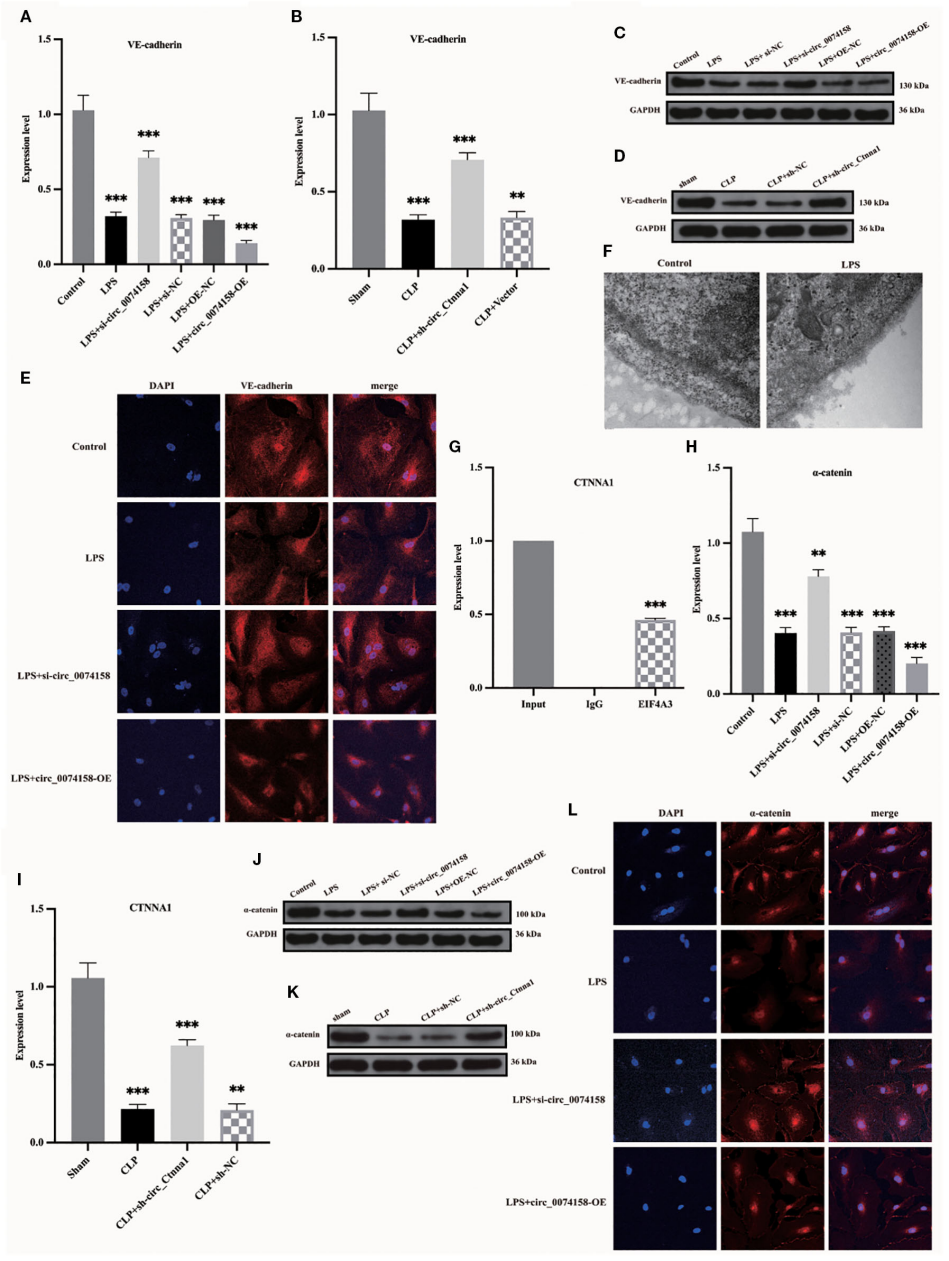
hsa_circ_0074158 decreases host gene CTNNA1 expression in sepsis. **(A, B)** RT-qPCR of animal experiments and cellular experiments showed low expression of VE-cadherin in sepsis. **(C, D)** Western blotting of animal experiments and cellular experiments showed low expression of VE-cadherin in sepsis. **(E)** IF assay showing low expression of VE-cadherin in sepsis. **(F)** Electron microscopy analysis showed that the glycocalyx is degraded in sepsis. bar=500 nm. **(G)** RIP experiments showed that EIF4A3 could interact with the host gene CTNNA1 of hsa_circ_0074158. **(H, I)** CTNNA1 encodes α-catenin, and RT-qPCR of animal experiments and cellular experiments showed that CTNNA1 is lowly expressed in sepsis. **(J)**, **(K)** Western blotting of animal experiments and cellular experiments showed low expression of VE-cadherin in sepsis. **(L)** IF experiments showed low expression of α-catenin in sepsis. ^***^ *p*<0.001, ^**^ *p*<0.01, ^*^ *p*<0.05.

The results of hsa_circ_0074158 mass spectrometry showed that hsa_circ_0074158 is combined with EIF4A3 (**Figure 3A**). EIF4A3 is an adenosine triphosphate-dependent RNA helicase, which is a core component of the exon-junction complex and plays an important role in RNA metabolism. After binding with circRNA, EIF4A3 can prevent the nuclear export of host gene mRNA and thus inhibit tumor metastasis, promote tumor progression by enhancing mRNA stability, and protect atherosclerotic plaque stability by inhibiting autophagy. In conclusion, EIF4A3 has important significance in the development and progression of diseases, but the correlation of EIF4A3 with endothelial barrier function in sepsis remains unclear. To screen proteins bound to hsa_circ_0074158, we performed RNA pull-down experiments and mass spectrometry on hsa_circ_0074158 and analyzed proteomic results. Gene Ontology (GO) showed that proteins interacting with hsa_circ_0074158 were distributed in the nucleus, cytoplasm, and cell junction, and can bind to DNA and RNA, and these proteins participate in biological processes such as DNA transcription and molecular function regulation (**Figures 3B-D**). Kyoto Encyclopedia of Genes and Genomes (KEGG) showed that the proteins interacting with hsa_circ_0074158 were mainly enriched in the pathways associated with infection (**Figure 4E**). Therefore, we hypothesized that EIF4A3 may be associated with the sepsis endothelial barrier function and may be involved in the regulation of the endothelial barrier function in sepsis.

### 3.4 hsa_circ_0074158 affects the endothelial barrier function in sepsis by regulating the stability of the host gene CTNNA1 (mRNA)

VE-cadherin and the integrity of the glycocalyx system, a crucial molecular component of endothelial barrier function, demonstrate significant impairment during the pathological process of sepsis, a notable alteration validated by various methodologies, including RT-qPCR, WB, and electron microscopy techniques (**Figures 4A-F**). Importantly, the RIP assay in preliminary experiments not only confirmed the molecular interactions between hsa_circ_0074158 and EIF4A3 but also revealed, for the first time, that EIF4A3 formed a specific binding with the mRNA of the host gene CTNNA1 (**Figure 4G**). Based on the findings that (i) hsa_circ_0074158 is derived from reverse splicing of the CTNNA1 gene; (ii) hsa_circ_0074158 significantly affects endothelial barrier function in a sepsis model; and (iii) the CTNNA1 gene encodes α-catenin, a key regulatory protein of the endothelial barrier, we propose the scientific hypothesis that hsa_circ_0074158 may regulate endothelial barrier function through EIF4A3. hsa_circ_0074158 could be involved in the pathological process of sepsis by modulating the expression of the host gene CTNNA1. Experimental data indicated that both mRNA and protein expression levels of CTNNA1 exhibited significant down-regulation in the sepsis group (P<0.01), and hsa_circ_0074158 was negatively correlated with CTNNA1 expression (**Figures 4H-L**). These findings suggest that hsa_circ_0074158 may inhibit the transcription of the host gene CTNNA1 through an epigenetic regulatory mechanism, exacerbating sepsis-associated endothelial barrier dysfunction.

### 3.5 EIF4A3 impairs endothelial barrier function in sepsis

**Figure 5:**
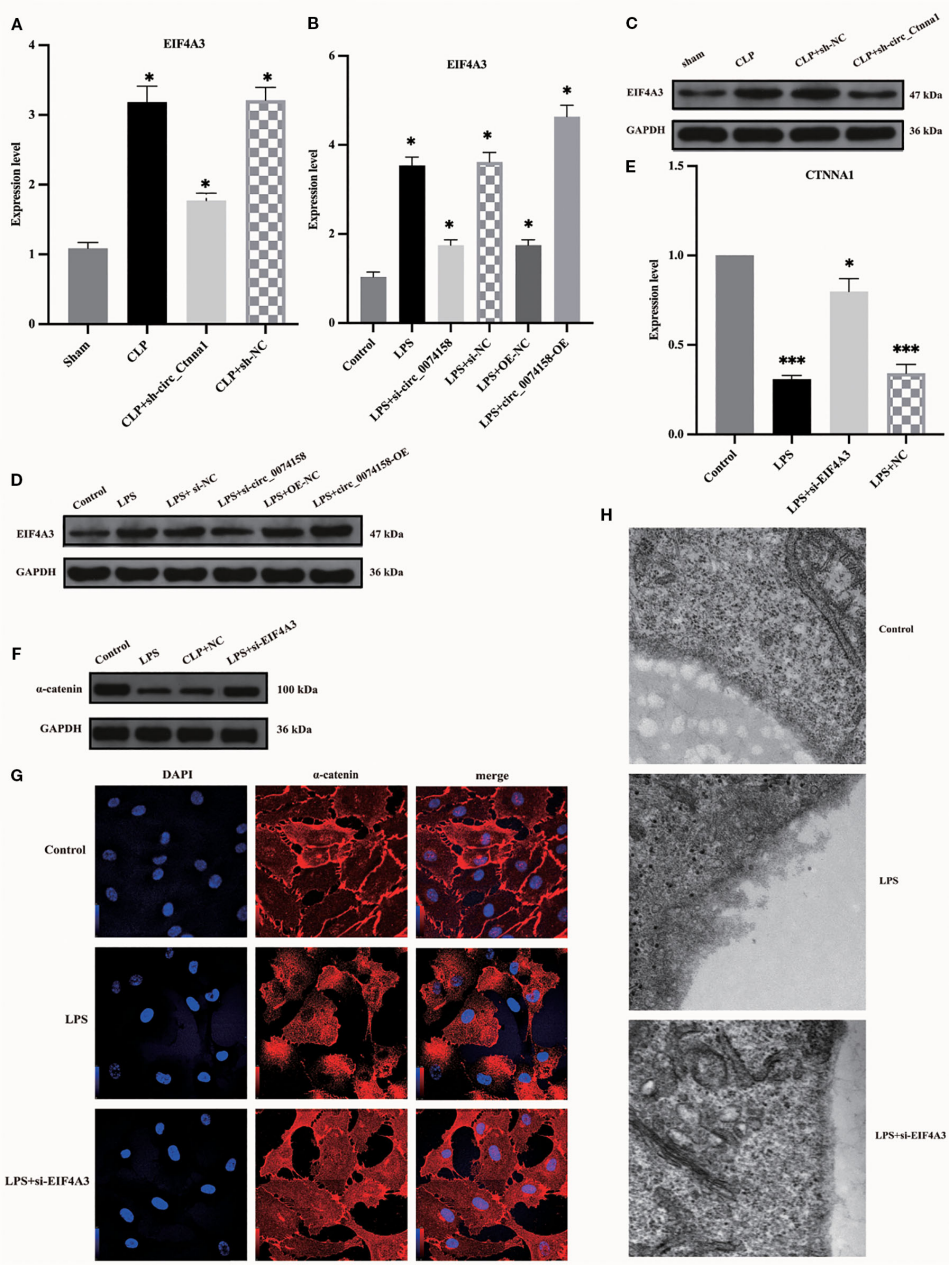
EIF4A3 impairs endothelial barrier function in sepsis. **(A, B)** RT-qPCR revealed upregulated EIF4A3 inversely correlated with circ_0074158 in sepsis models. **(C, D)** Western blotting confirmed the elevated protein levels of EIF4A3 under septic conditions. **(E-G)** Targeted knockdown of EIF4A3 in sepsis models resulted in substantial restoration of CTNNA1 expression at both transcriptional (mRNA) and translational (protein) levels. **(H)** Electron microscopy analysis revealed marked attenuation of glycocalyx degradation following EIF4A3 suppression in septic conditions. ^***^ *p*<0.001, ^**^ *p*<0.01, ^*^ *p*<0.05.

Our previous investigation revealed distinct expression patterns of hsa_circ_0074158 and its associated molecules in sepsis pathogenesis. Specifically, hsa_circ_0074158 showed significant upregulation in sepsis (8), while the transcriptional activity of its host gene CTNNA1 demonstrated concurrent suppression (**Figures 4H, I**). Complementary analyses using RT-qPCR and immunoblotting further identified a notable increase in EIF4A3 expression in the sepsis cohort, with a strong positive correlation observed between circ_0074158 and EIF4A3 levels (**Figures 5A-D**). RIP experiments confirmed a direct molecular interaction between EIF4A3 and hsa_circ_0074158 (**Figure 4G**), suggesting functional interplay within this regulatory axis. To delineate the mechanistic significance of the hsa_circ_0074158/EIF4A3/CTNNA1 axis, we employed siRNA-mediated EIF4A3 knockdown followed by comprehensive phenotypic assessments. Subsequent analyses revealed a significant recovery of CTNNA1 expression at both transcriptional and translational levels after intervention (**Figures 5E-G**). Notably, EIF4A3 suppression reduced sepsis-induced glycocalyx degradation (**Figure 5H**). Collectively, these findings establish a pathogenic cascade wherein: (i) sepsis-induced hsa_circ_0074158 overexpression exacerbates endothelial barrier dysfunction; (ii) concomitant EIF4A3 upregulation synergistically worsens vascular compromise; and (iii) bidirectional molecular interactions between these effectors create a self-amplifying circuit driving disease progression.

### 3.6 hsa_circ_0074158 affects the endothelial barrier function in sepsis by regulating the host gene CTNNA1 (mRNA) stability

**Figure 6:**
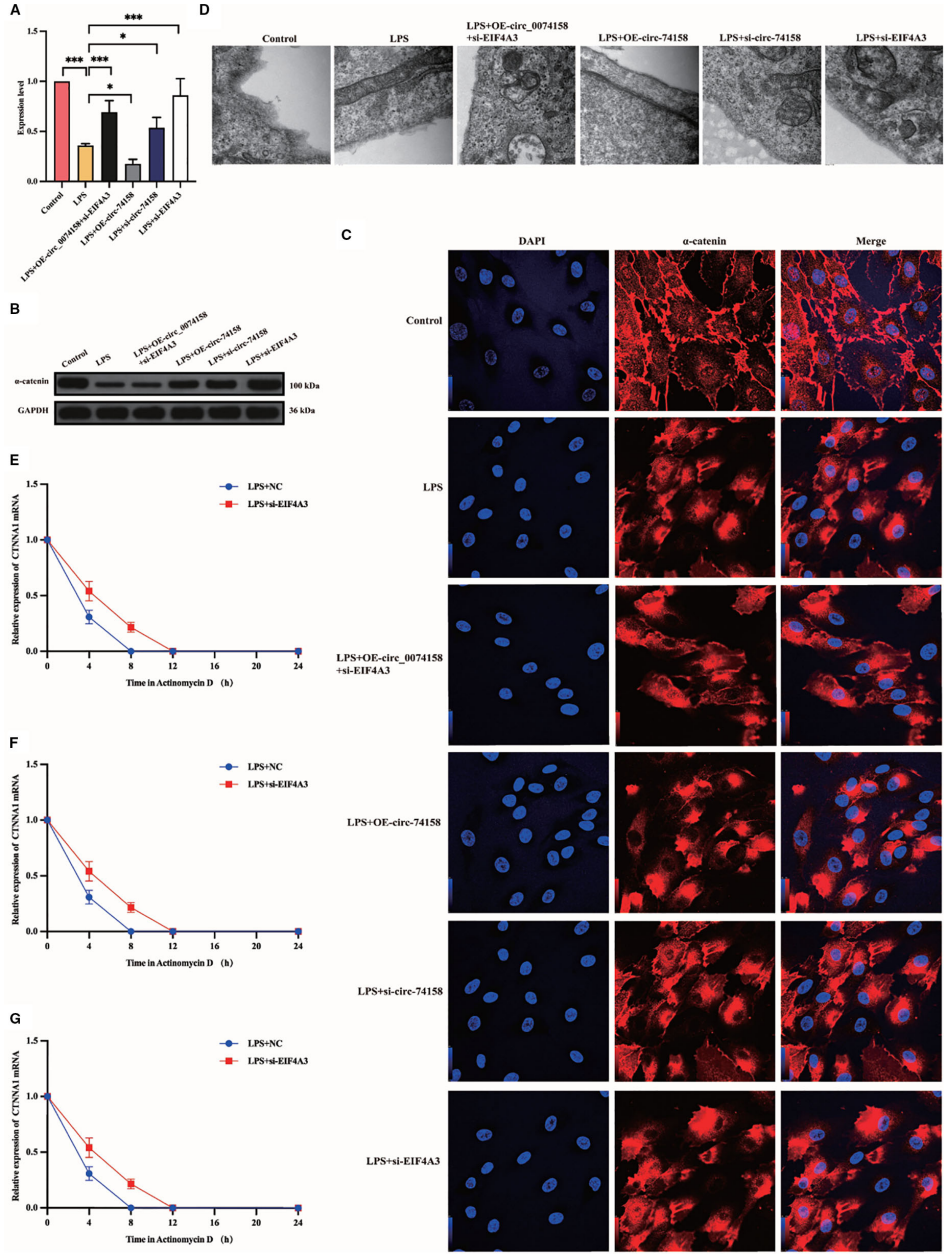
hsa_circ_0074158 compromises the endothelial barrier in sepsis via destabilizing CTNNA1 mRNA. **(A-D)** Rescue experiment demonstrates that EIF4A3 knockdown mechanistically rescued the hsa_circ_0074158-mediated suppression of its host gene CTNNA1, thereby ameliorating sepsis-induced endothelial barrier dysfunction. **(E–G)** After actinomycin D treatment, the mRNA stability of CTNNA1 in sepsis was determined by RT-qPCR. ^***^ *p*<0.001, ^**^ *p*<0.01, ^*^ *p*<0.05, ^ns^ *p*>0.05.

**Figure 7:**
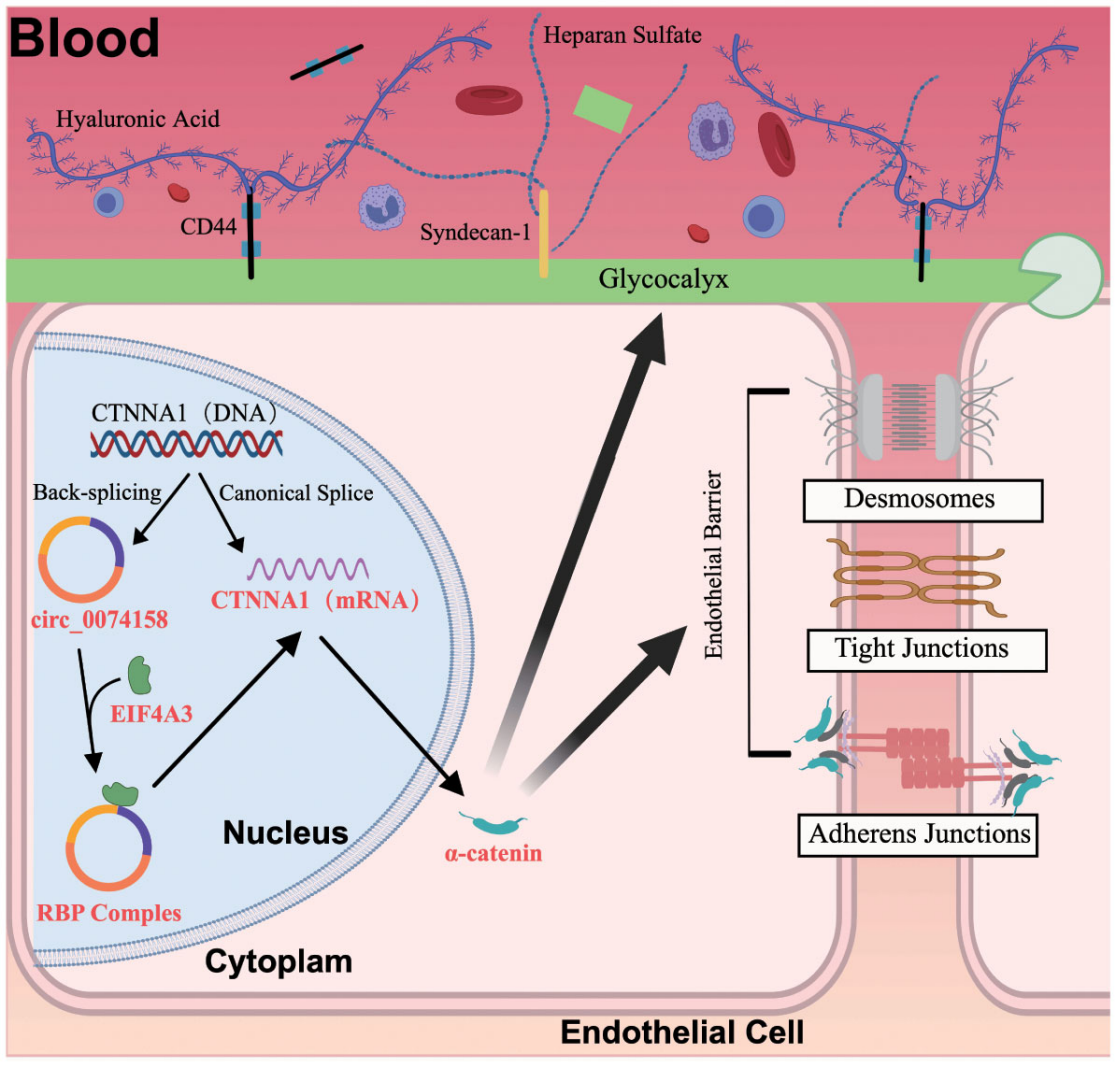
The hsa_circ_0074158/EIF4A3/CTNNA1 axis mechanism in sepsis.

To delineate the regulatory mechanism of the hsa_circ_0074158/EIF4A3 axis on its host gene CTNNA1, we established the following genetic perturbation models in HUVECs: si-circ_0074158, OE-circ_0074158, and si-EIF4A3. Multidimensional validation through RT-qPCR and WB demonstrated that hsa_circ_0074158 interacts with EIF4A3 to modulate sepsis-induced endothelial barrier dysfunction by regulating CTNNA1. Rescue experiment design: (i) Control group; (ii) LPS group; (iii) LPS + OE-circ_0074158 + si-EIF4A3; (iv) LPS + OE-circ_0074158; (v) LPS + si-circ_0074158; (vi) LPS + si-EIF4A3. RT-qPCR results indicated that in sepsis, compared to the LPS group, the expression of CTNNA1 was up-regulated in the LPS + OE-circ_0074158 + si-EIF4A3 group, and down-regulated in the LPS + OE-circ_0074158 group. The expression of CTNNA1 was also up-regulated in the LPS + si-circ_0074158 group, and in the LPS + si-EIF4A3 group, with the differences being statistically significant (**Figure 6A**). The expression was further confirmed by WB, IF and electron microscopy (**Figures 6B-D**). To assess the post-transcriptional regulation of CTNNA1, actinomycin D was added to HUVECs subjected to circ_0068631 knockdown (si-circ_0068631), hsa_circ_0074158 overexpression (OE-circ_0074158), and EIF4A3 knockdown (si-EIF4A3). Notably, CTNNA1 mRNA stability increased due to hsa_circ_0074158 depletion or EIF4A3 depletion and decreased due to hsa_circ_0074158 overexpression (**Figures 6E-G**). The mechanism of the hsa_circ_0074158/EIF4A3/CTNNA1 axis in sepsis is depicted in **Figure 7**.

## 4 Discussion

Despite advances in health care, sepsis remains a major global health burden with high morbidity and mortality rates (24). The pathogenesis of sepsis is complex, with vascular endothelial barrier damage being a key factor. The target therapy of sepsis has become an area of intense research (12). circRNAs are involved in the regulation of the sepsis pathogenesis (7). Our previous studies have shown the important role of hsa_circ_0074158 in regulating the endothelial barrier function in sepsis (8), and this study found that hsa_circ_0074158 affects the endothelial barrier function in sepsis by regulating its host gene CTNNA1 (mRNA) and forming an RBP complex with EIF4A3 to damage the endothelial barrier function.

In this study, we have made significant progress in understanding the role of hsa_circ_0074158 in sepsis. We found that hsa_circ_0074158 regulates the endothelial barrier function in sepsis by modulating its host gene CTNNA1 (mRNA). Additionally, hsa_circ_0074158 forms an RBP complex with RNA-binding protein EIF4A3, which reduces the stability of CTNNA1 (mRNA) and decreases the production of α-catenin, thereby impairing the endothelial barrier function in sepsis. EIF4A3 is an adenosine triphosphate-dependent RNA helicase, which is a core component of the exon-junction complex and plays an important role in RNA metabolism (11, 14, 22, 23). However, the correlation of EIF4A3 with endothelial barrier function in sepsis remains unclear. To screen proteins bound to hsa_circ_0074158, we performed RNA pull-down experiments and mass spectrometry hsa_circ_0074158 and analyzed proteomic results. Integrating GO and KEGG pathway analyses, we postulated that EIF4A3 might be implicated in sepsis-induced endothelial barrier dysfunction. This hypothesis was experimentally validated through comprehensive functional studies, including gain- and loss-of-function experiments. This study provides the first evidence that hsa_circ_0074158, a circular RNA generated from the CTNNA1 locus, exacerbates sepsis-induced endothelial barrier failure through an EIF4A3-dependent mRNA destabilization mechanism. CircRNA as a master regulator of host gene expression is contrary to previous reports of circRNAs stabilizing host transcripts via miRNA sponging (11), our data reveal hsa_circ_0074158 suppresses CTNNA1 through competitive binding with EIF4A3—a component of the exon junction complex (EJC). This paradoxical regulation may reflect tissue-specific EJC dynamics, as sepsis-induced inflammation alters RNA-binding protein stoichiometry (22). The observed glycocalyx preservation via EIF4A3 inhibition suggests RNA metabolism directly regulates endothelial surface architecture. This expands the paradigm of endothelial barrier regulation beyond classical cytoskeletal remodeling.

However, our study has some limitations. Although we have demonstrated the mechanisms involved to some extent, further research is required to fully understand the complex interactions and signaling pathways in sepsis. Future studies should focus on exploring potential therapeutic strategies based on these findings to improve the prognosis for sepsis patients. Additionally, determining the exact structural basis for hsa_circ_0074158-EIF4A3 interaction (such as AU-rich element recognition) will require crystallographic analysis. While our CLP model replicates the hyperpermeability seen in human sepsis, species differences in circRNA processing necessitate validation in non-human primates. Although mouse circ_Ctnna1 and human hsa_circ_0074158 show high homology, this partial conservation may limit direct application to human diseases due to interspecies differences, and further studies using humanized models are needed. We have acknowledged that more preclinical studies are essential before fully evaluating the therapeutic potential. Currently, our multi-center study is ongoing, and we believe these complementary approaches will provide a necessary bridge between our current findings and potential clinical applications.

​In summary, our findings delineate a novel pathogenic axis wherein hsa_circ_0074158 impairs endothelial barrier integrity during sepsis by recruiting the RNA-binding protein EIF4A3. This circRNA-protein complex post-transcriptionally destabilizes host gene CTNNA1 (mRNA) of hsa_circ_0074158. Crucially, rescue experiments demonstrated the mechanistic hierarchy of this regulatory cascade. These results not only advance our understanding results not only advance our understanding of sepsis-associated vascular leakage but also highlight the hsa_circ_0074158/EIF4A3 complex as a promising therapeutic node for pharmacological intervention.

## Data Availability

The raw data supporting the conclusions of this article will be made available by the authors, without undue reservation.
