# Supplementary material for: Mechanistic study of the hsa_circ_0074158 binding EIF4A3 impairing sepsis-induced endothelial barrier
Source: Front Immunol. 2025 Sep 22;16:1621095. doi: 10.3389/fimmu.2025.1621095 (PMC12497631; doi:10.3389/fimmu.2025.1621095)
Supplement: Supplementary file 1 [file DataSheet1.docx]

Supplementary Figure 1：EIF4A3 may not only interact with hsa_circ_0074158 but

also affect the generation of hsa_circ_0074158.

Supplementary Table 1: There are 20 human circRNAs and 2 mouse homolog circRNAs associated with CTNNA1.
