# Supplementary figures and images for: Mechanistic study of the hsa_circ_0074158 binding EIF4A3 impairing sepsis-induced endothelial barrier
Source: Front Immunol. 2025 Sep 22;16:1621095. doi: 10.3389/fimmu.2025.1621095 (PMC12497631; doi:10.3389/fimmu.2025.1621095)

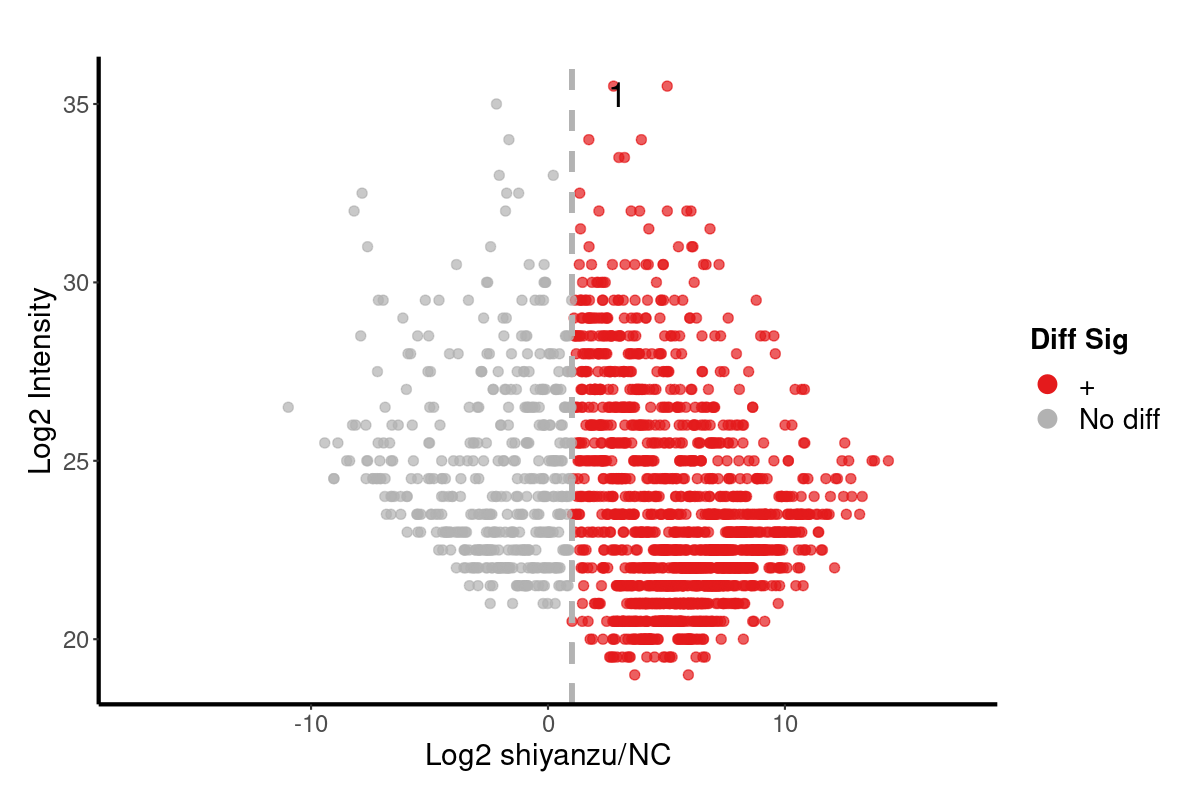

Supplement: Supplementary file 3 [file DataSheet3.zip › Fig. 3 GO and KEGG enrichment analysis of proteins identified by mass spectrometry./1.identification/1.5 ratio.volcano.png]

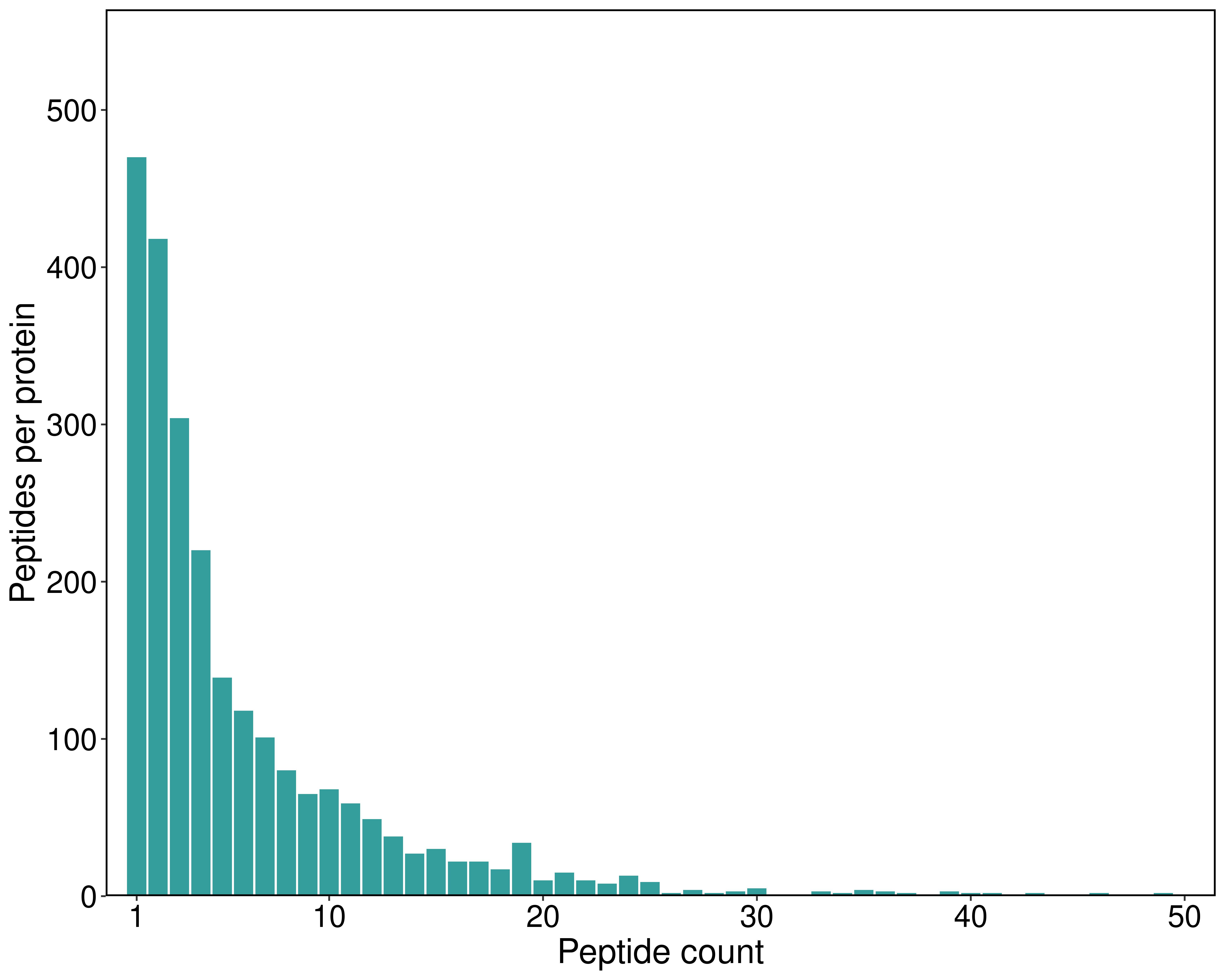

Supplement: Supplementary file 3 [file DataSheet3.zip › Fig. 3 GO and KEGG enrichment analysis of proteins identified by mass spectrometry./1.identification/1.2 protein_peptide.bar.png]

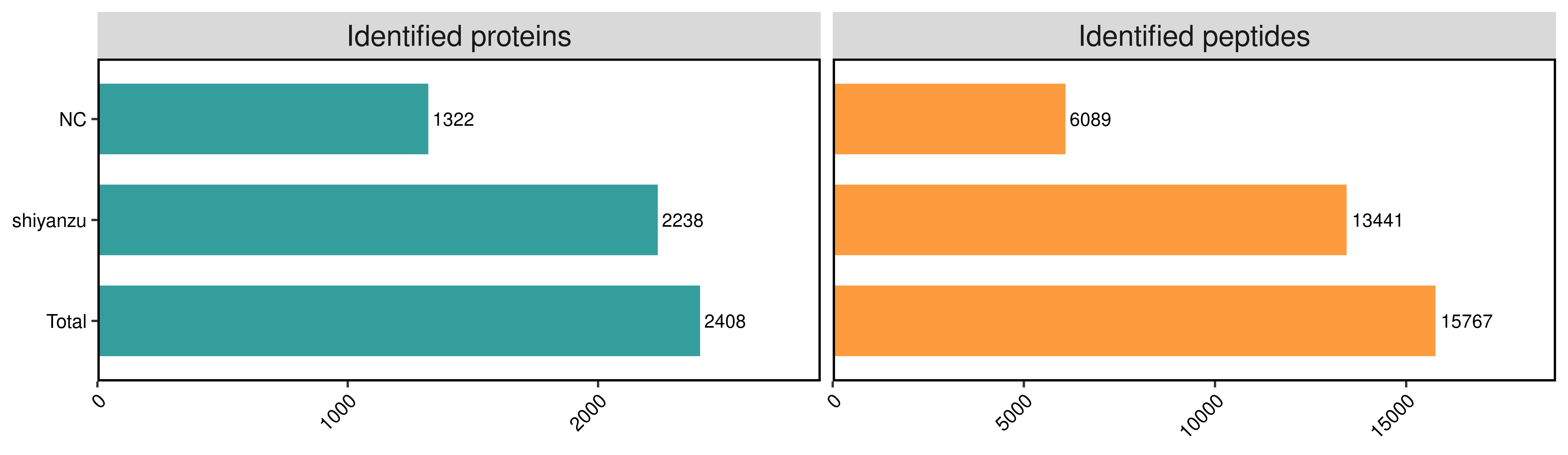

Supplement: Supplementary file 3 [file DataSheet3.zip › Fig. 3 GO and KEGG enrichment analysis of proteins identified by mass spectrometry./1.identification/1.4-1 proteins_stat.bar.png]

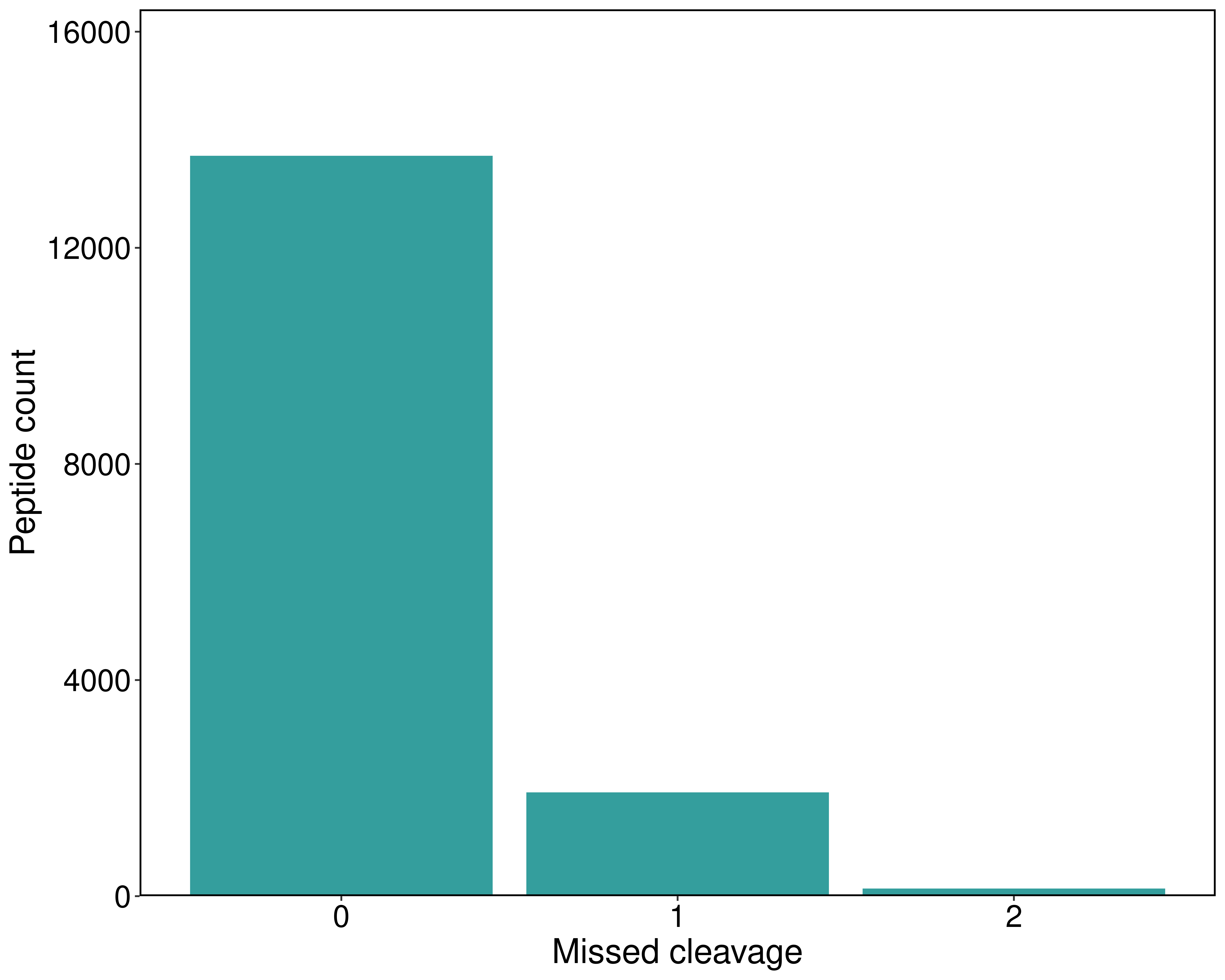

Supplement: Supplementary file 3 [file DataSheet3.zip › Fig. 3 GO and KEGG enrichment analysis of proteins identified by mass spectrometry./1.identification/1.3 missing_cleavage.bar.png]

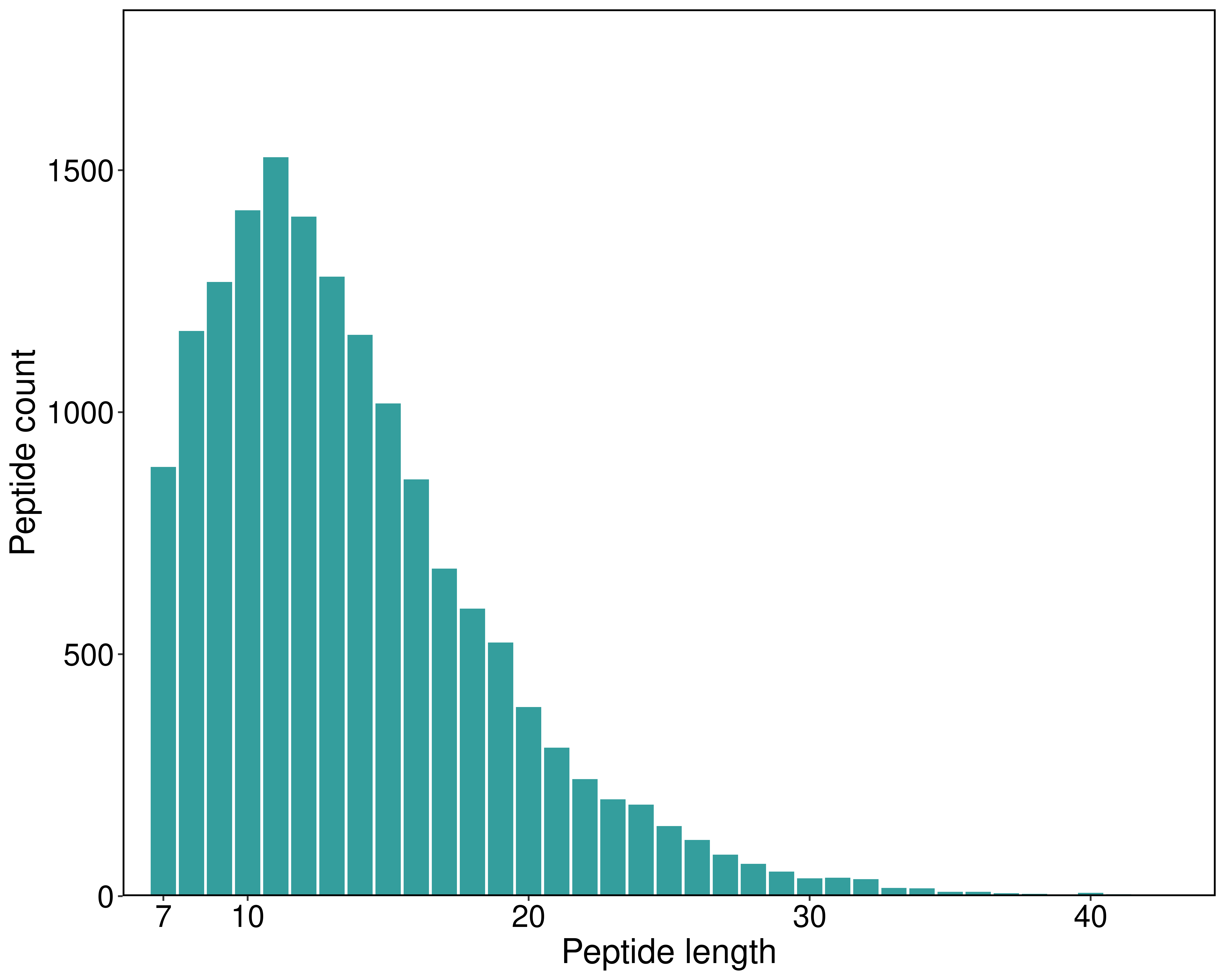

Supplement: Supplementary file 3 [file DataSheet3.zip › Fig. 3 GO and KEGG enrichment analysis of proteins identified by mass spectrometry./1.identification/1.1 peptide_length.bar.png]

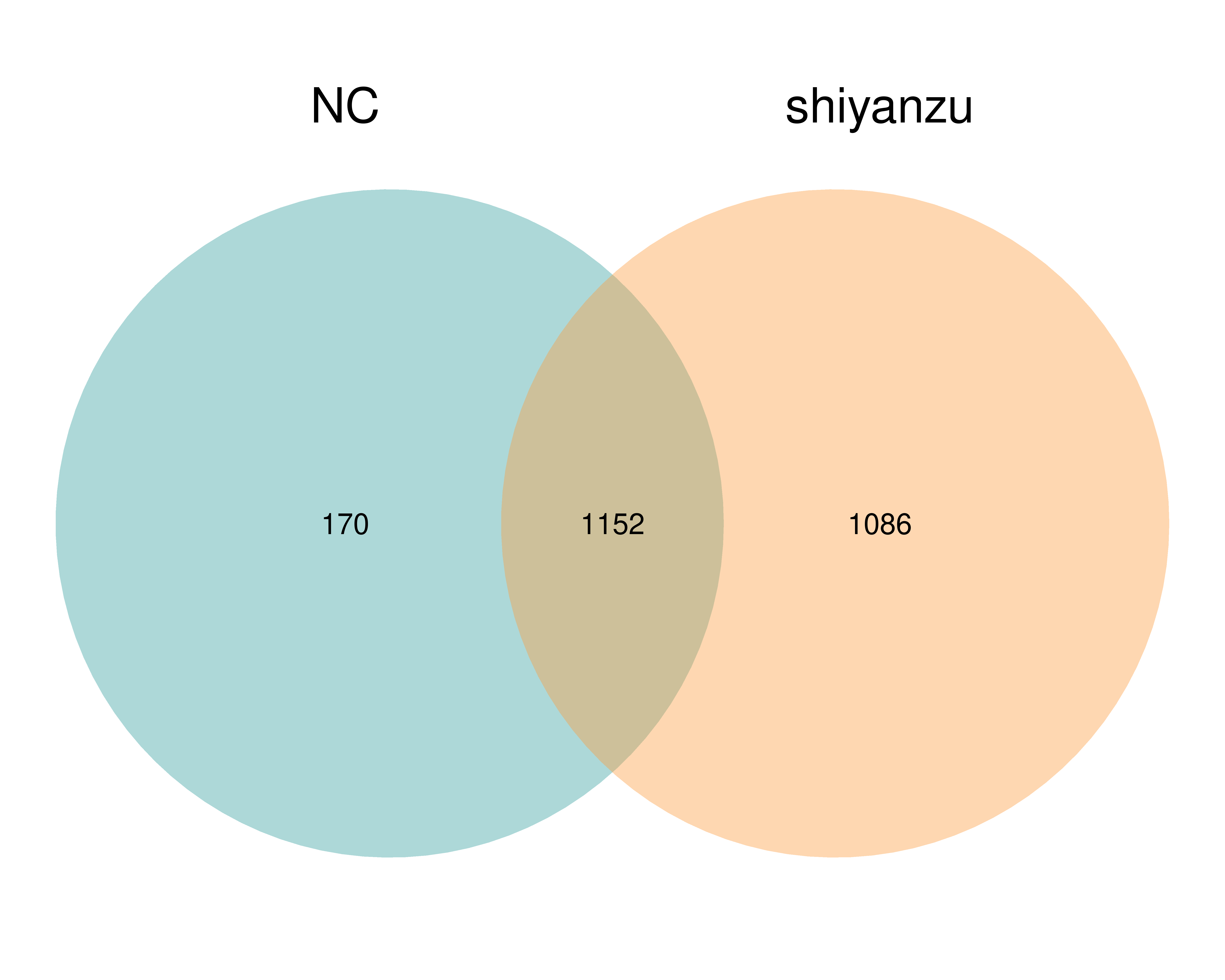

Supplement: Supplementary file 3 [file DataSheet3.zip › Fig. 3 GO and KEGG enrichment analysis of proteins identified by mass spectrometry./1.identification/1.4-2 proteins_stat.venn.png]

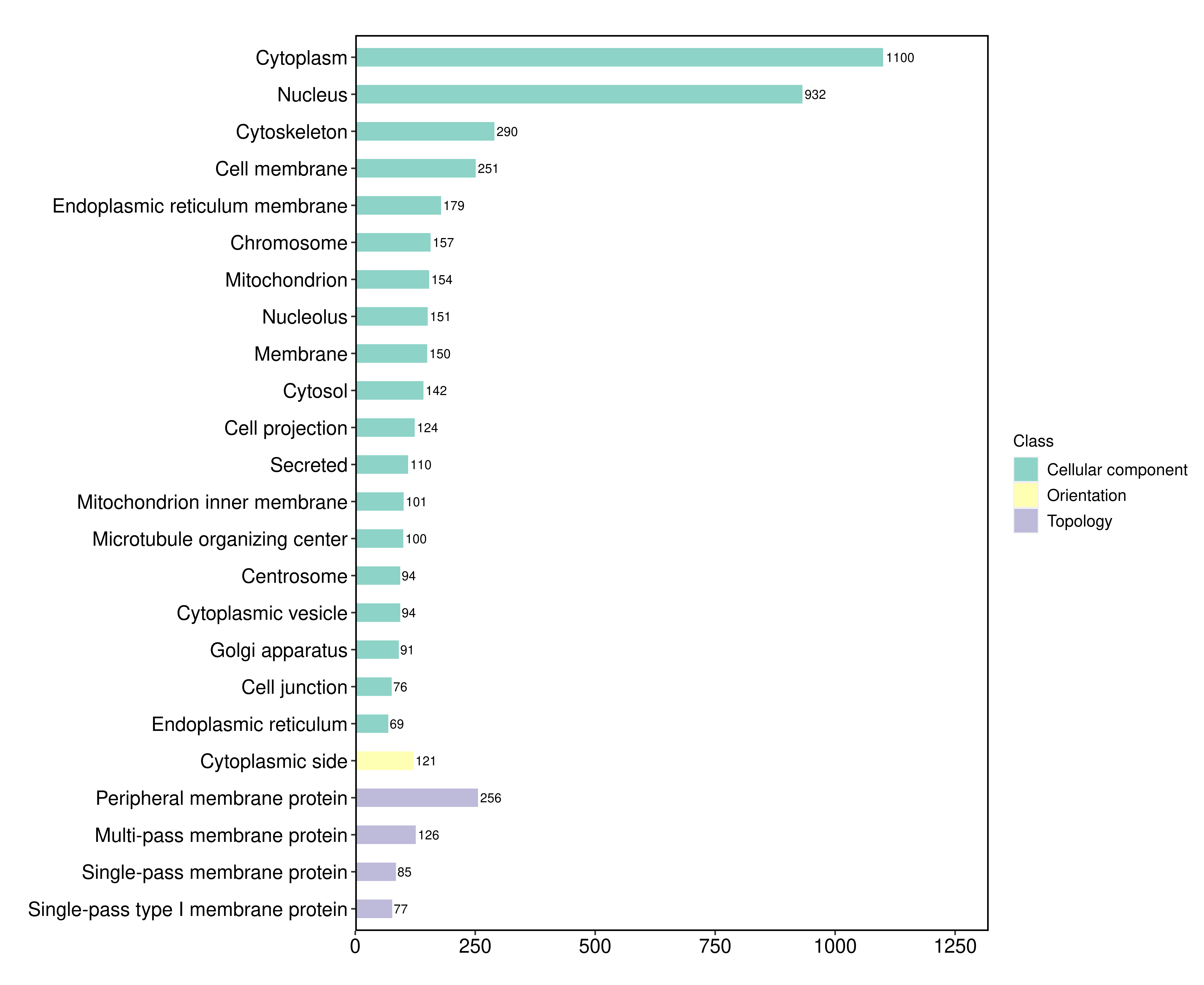

Supplement: Supplementary file 3 [file DataSheet3.zip › Fig. 3 GO and KEGG enrichment analysis of proteins identified by mass spectrometry./2.annotation/2.4 SL_annotation.bar.png]

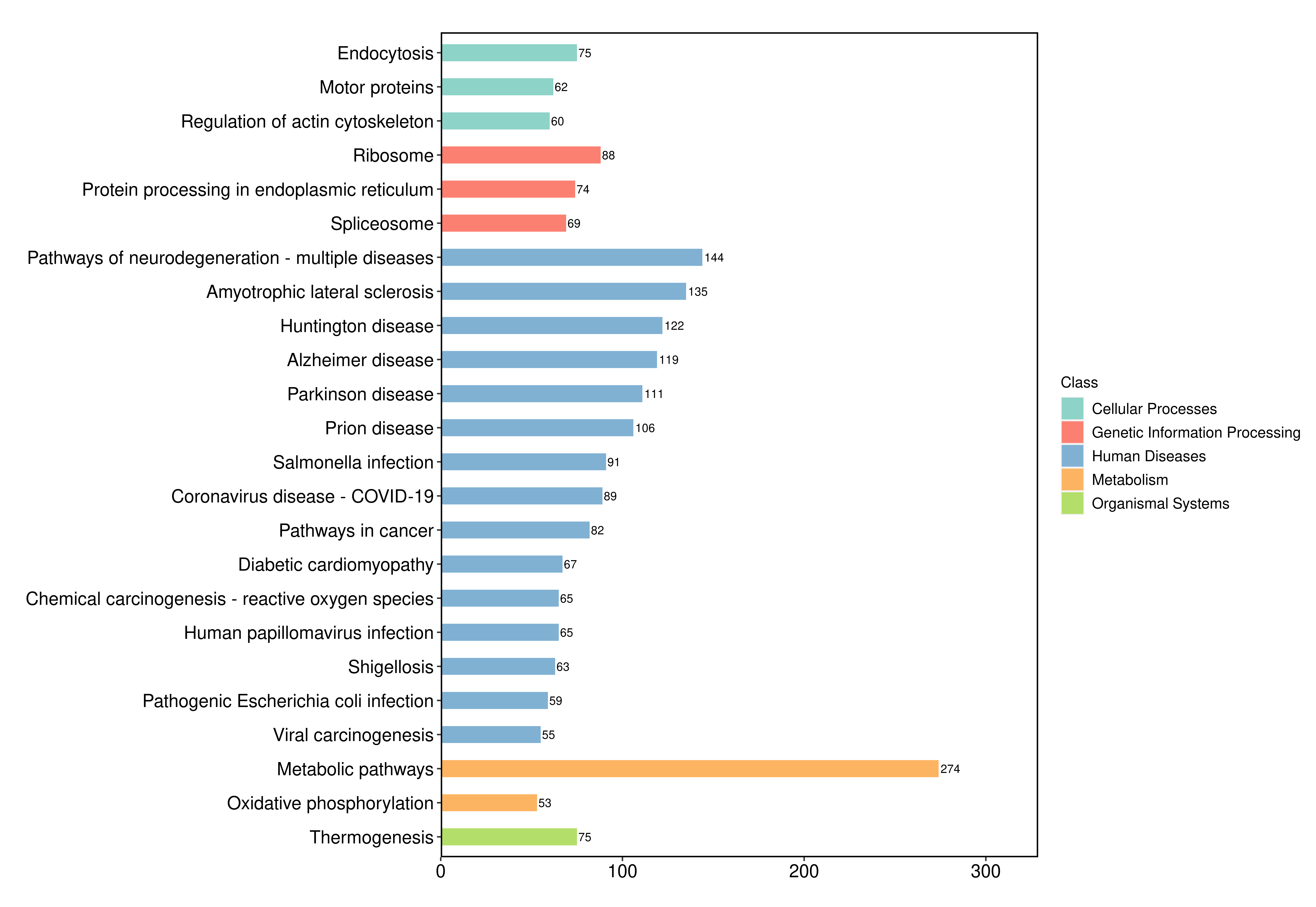

Supplement: Supplementary file 3 [file DataSheet3.zip › Fig. 3 GO and KEGG enrichment analysis of proteins identified by mass spectrometry./2.annotation/2.2 KEGG_annotation.bar.png]

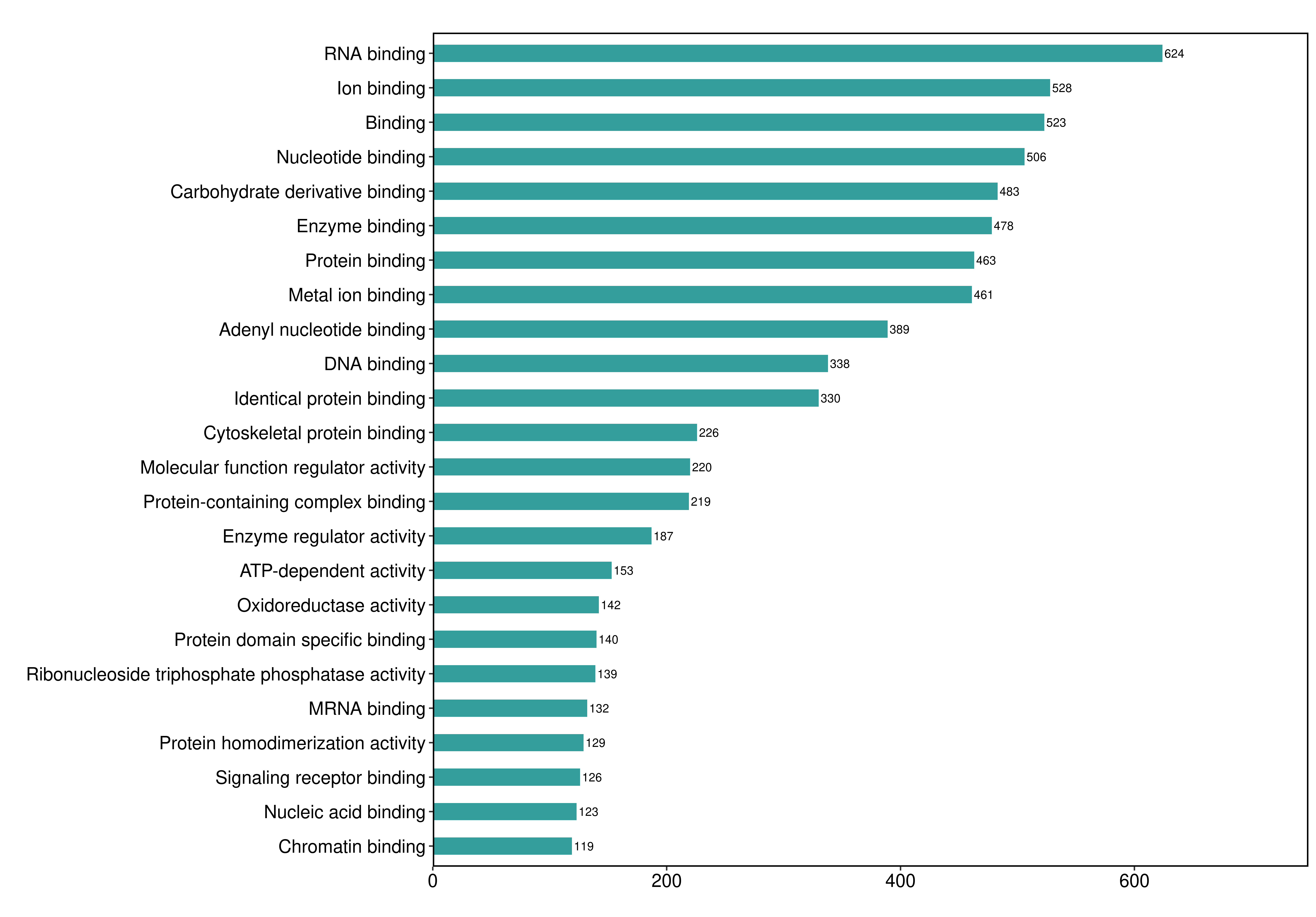

Supplement: Supplementary file 3 [file DataSheet3.zip › Fig. 3 GO and KEGG enrichment analysis of proteins identified by mass spectrometry./2.annotation/2.1-3 GOMF_annotation.bar.png]

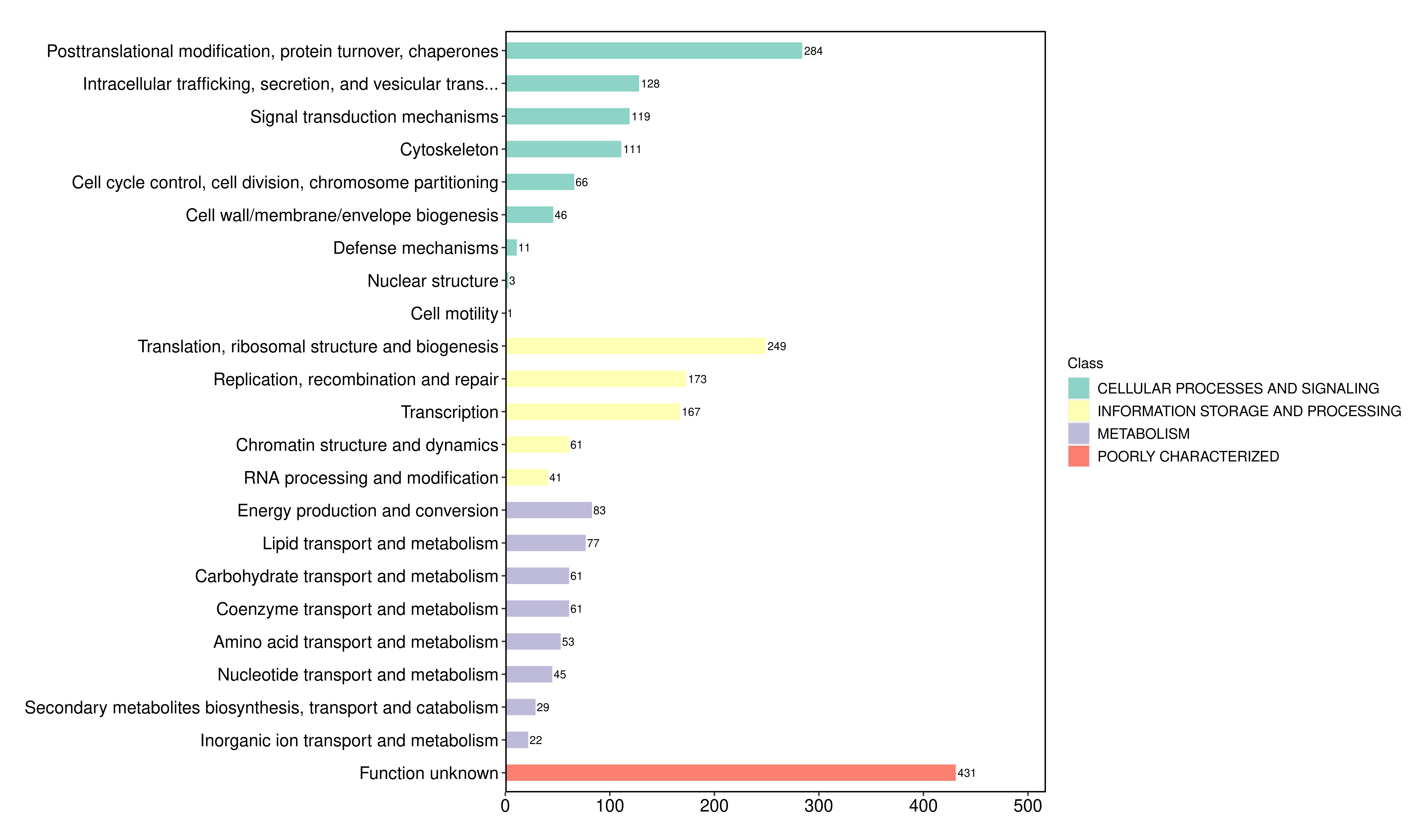

Supplement: Supplementary file 3 [file DataSheet3.zip › Fig. 3 GO and KEGG enrichment analysis of proteins identified by mass spectrometry./2.annotation/2.3 COG_annotation.bar.png]

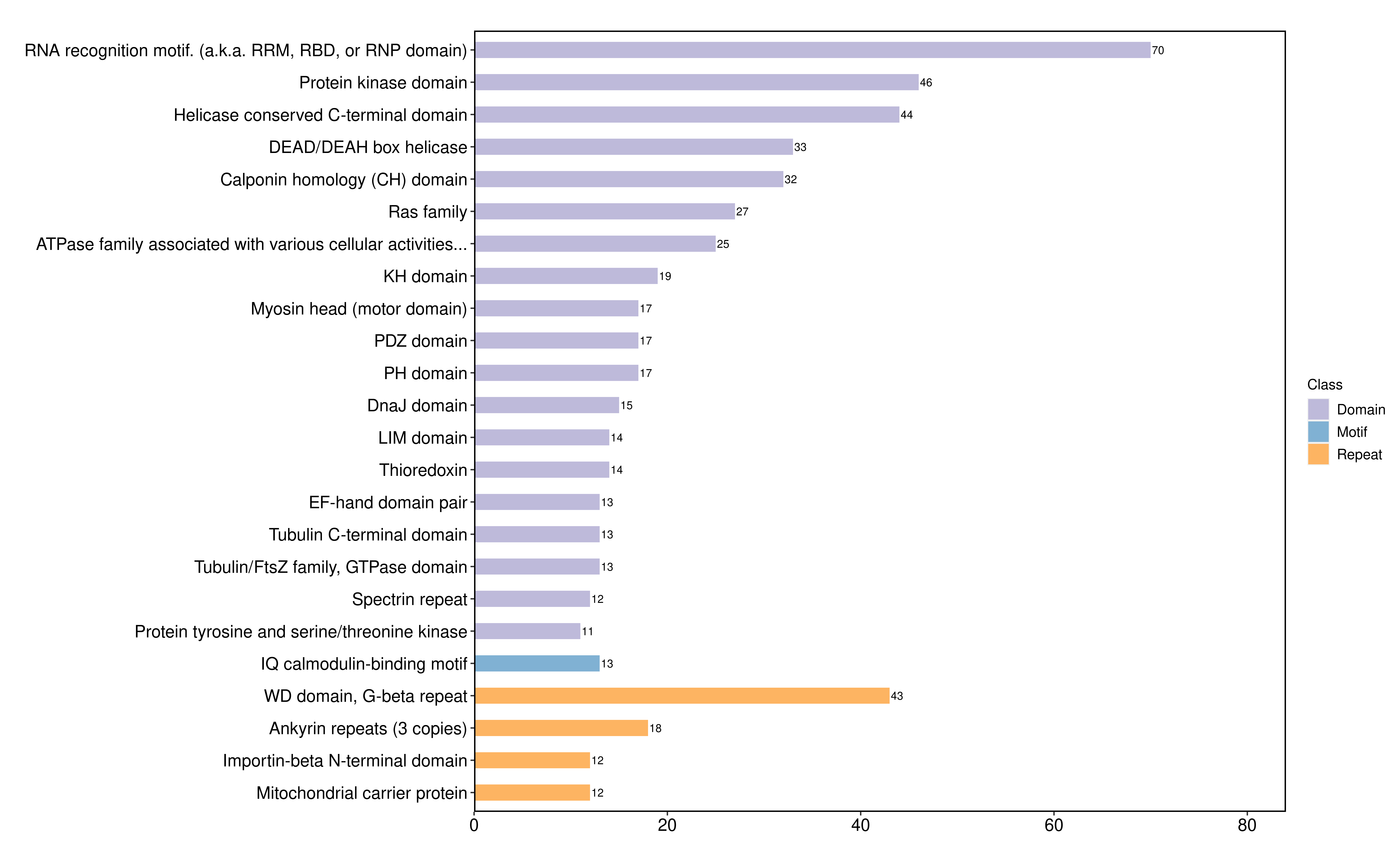

Supplement: Supplementary file 3 [file DataSheet3.zip › Fig. 3 GO and KEGG enrichment analysis of proteins identified by mass spectrometry./2.annotation/2.5 Pfam_annotation.bar.png]

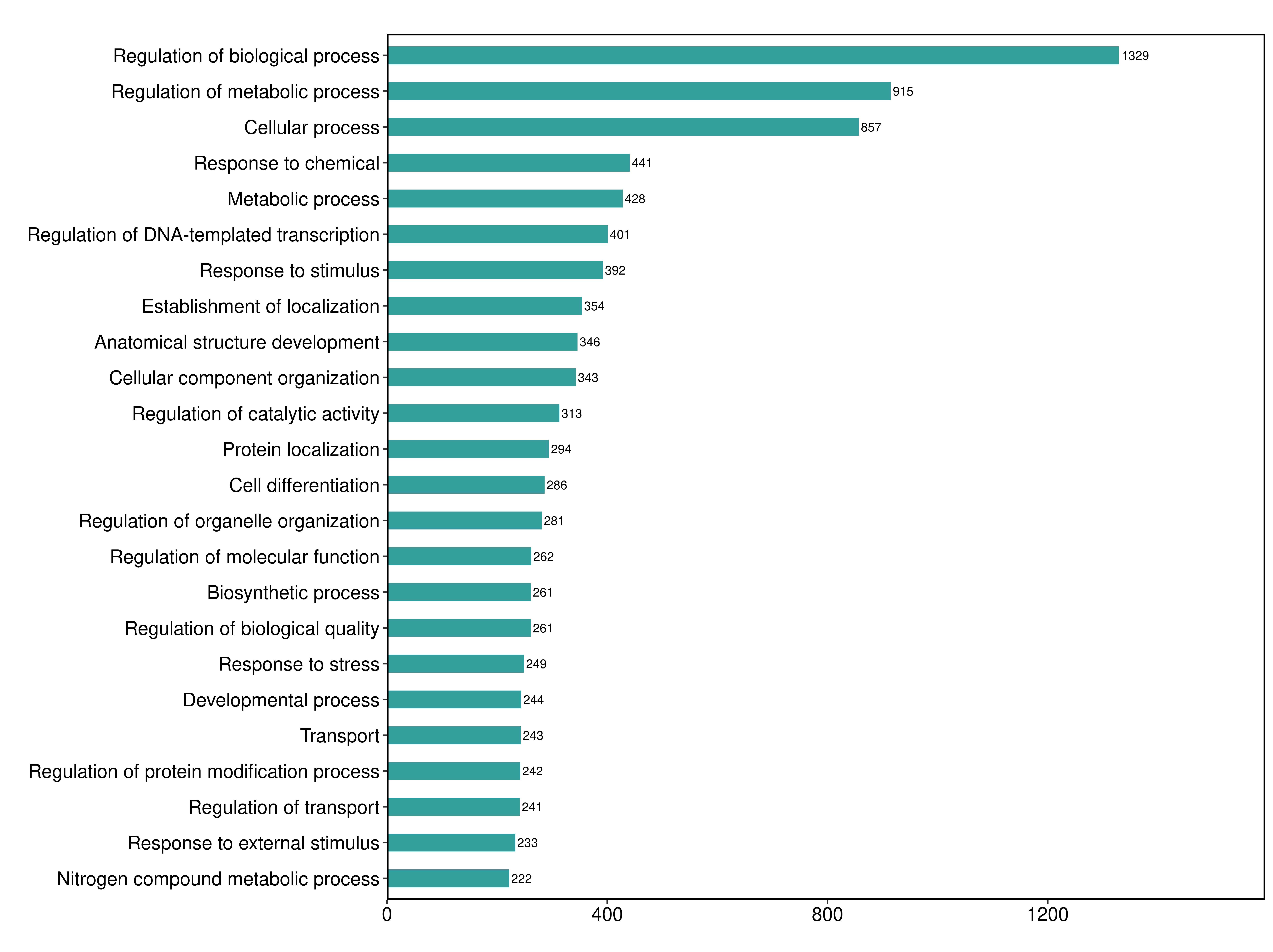

Supplement: Supplementary file 3 [file DataSheet3.zip › Fig. 3 GO and KEGG enrichment analysis of proteins identified by mass spectrometry./2.annotation/2.1-1 GOBP_annotation.bar.png]

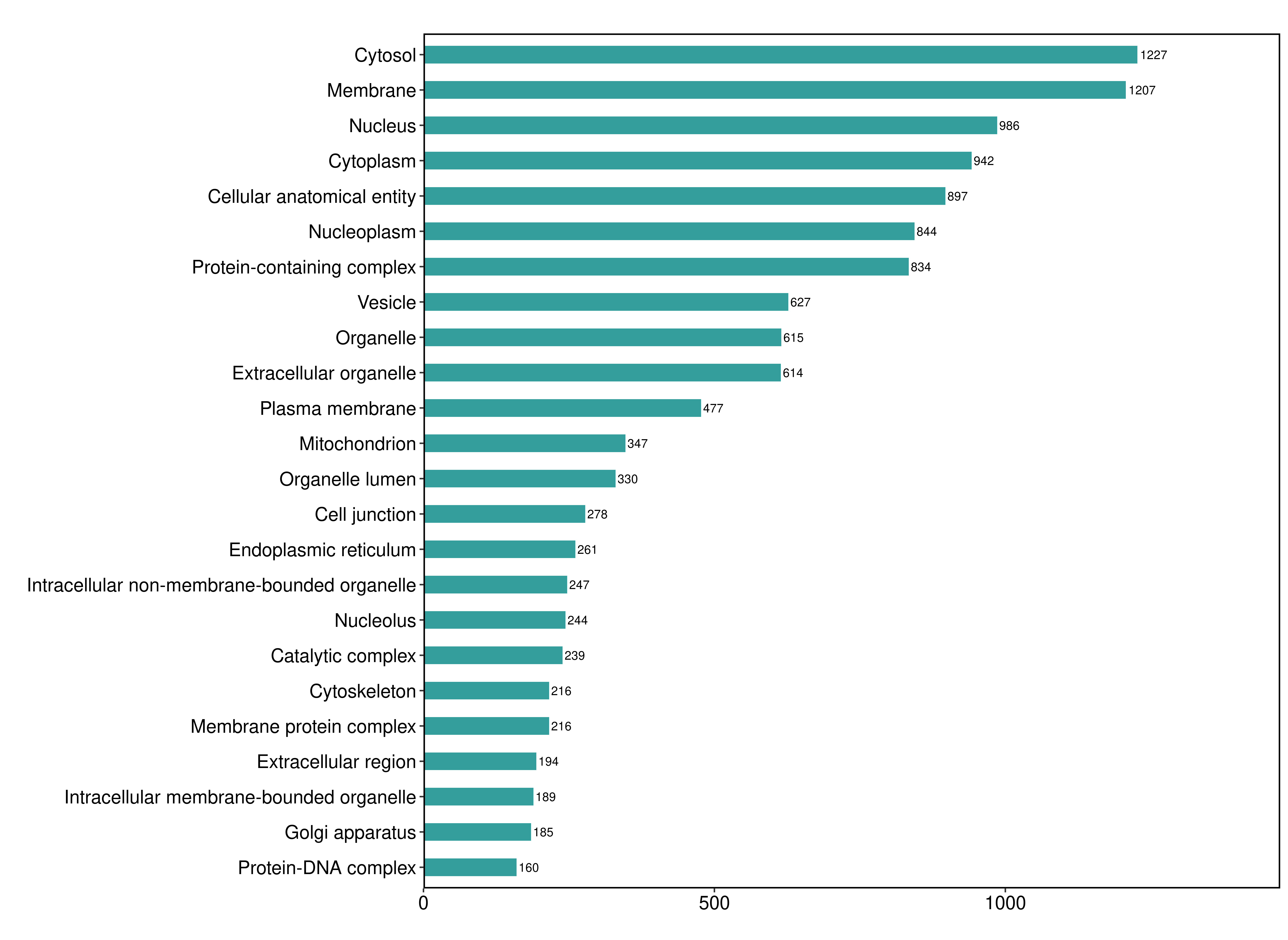

Supplement: Supplementary file 3 [file DataSheet3.zip › Fig. 3 GO and KEGG enrichment analysis of proteins identified by mass spectrometry./2.annotation/2.1-2 GOCC_annotation.bar.png]

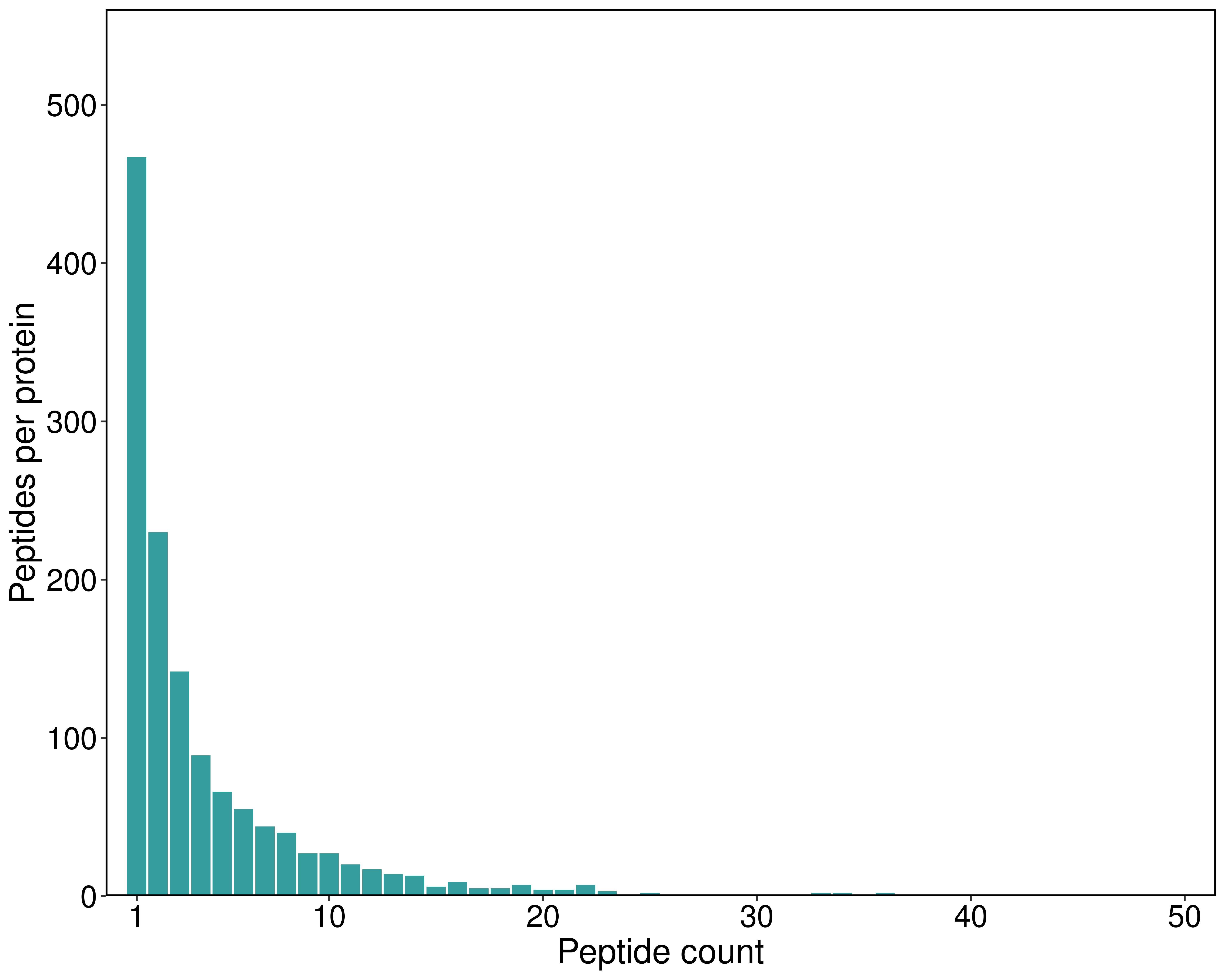

Supplement: Supplementary file 3 [file DataSheet3.zip › Fig. 3 GO and KEGG enrichment analysis of proteins identified by mass spectrometry./supplementary/NC/1.identification/1.2 protein_peptide.bar.png]

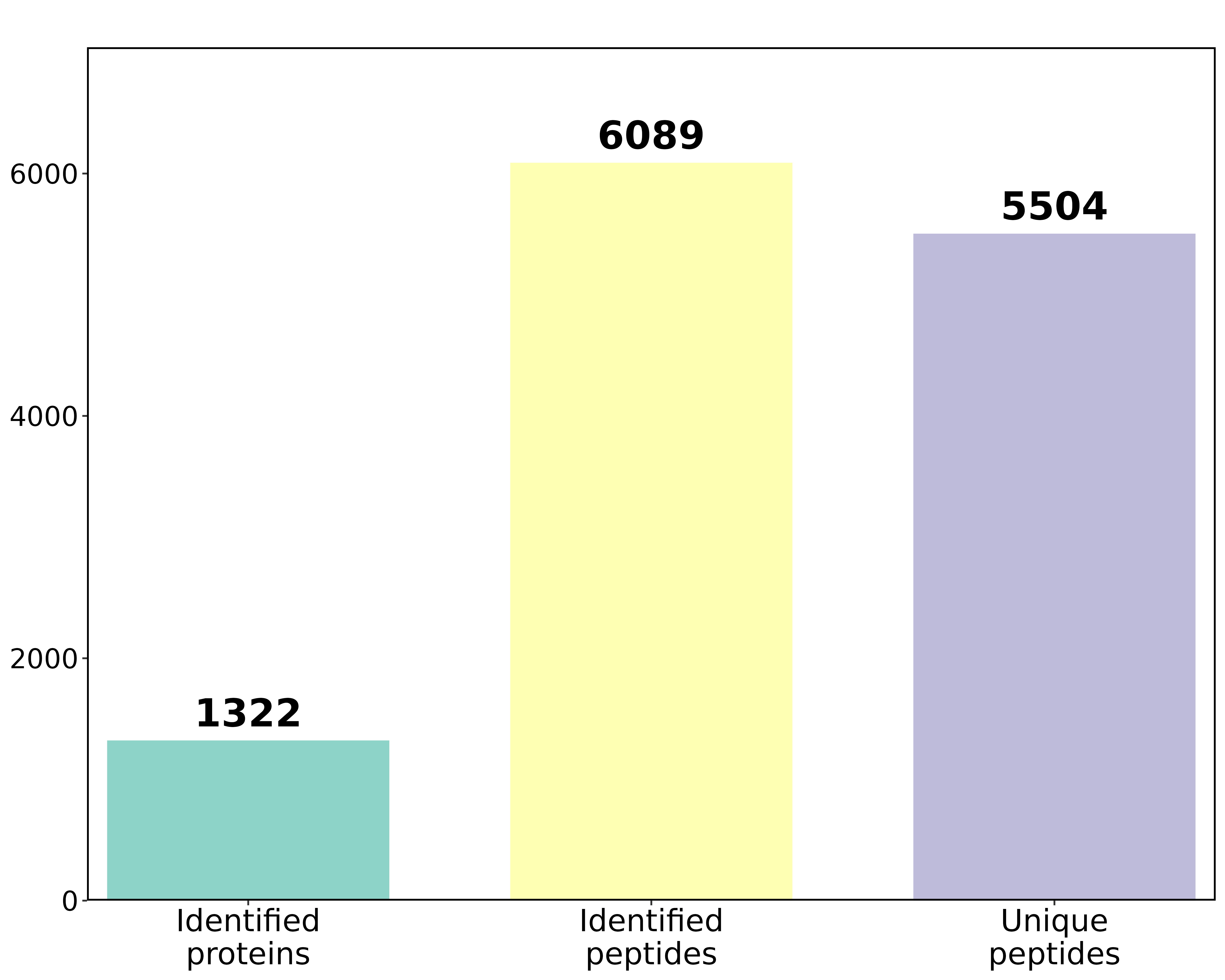

Supplement: Supplementary file 3 [file DataSheet3.zip › Fig. 3 GO and KEGG enrichment analysis of proteins identified by mass spectrometry./supplementary/NC/1.identification/1.4 proteins_stat.bar.png]

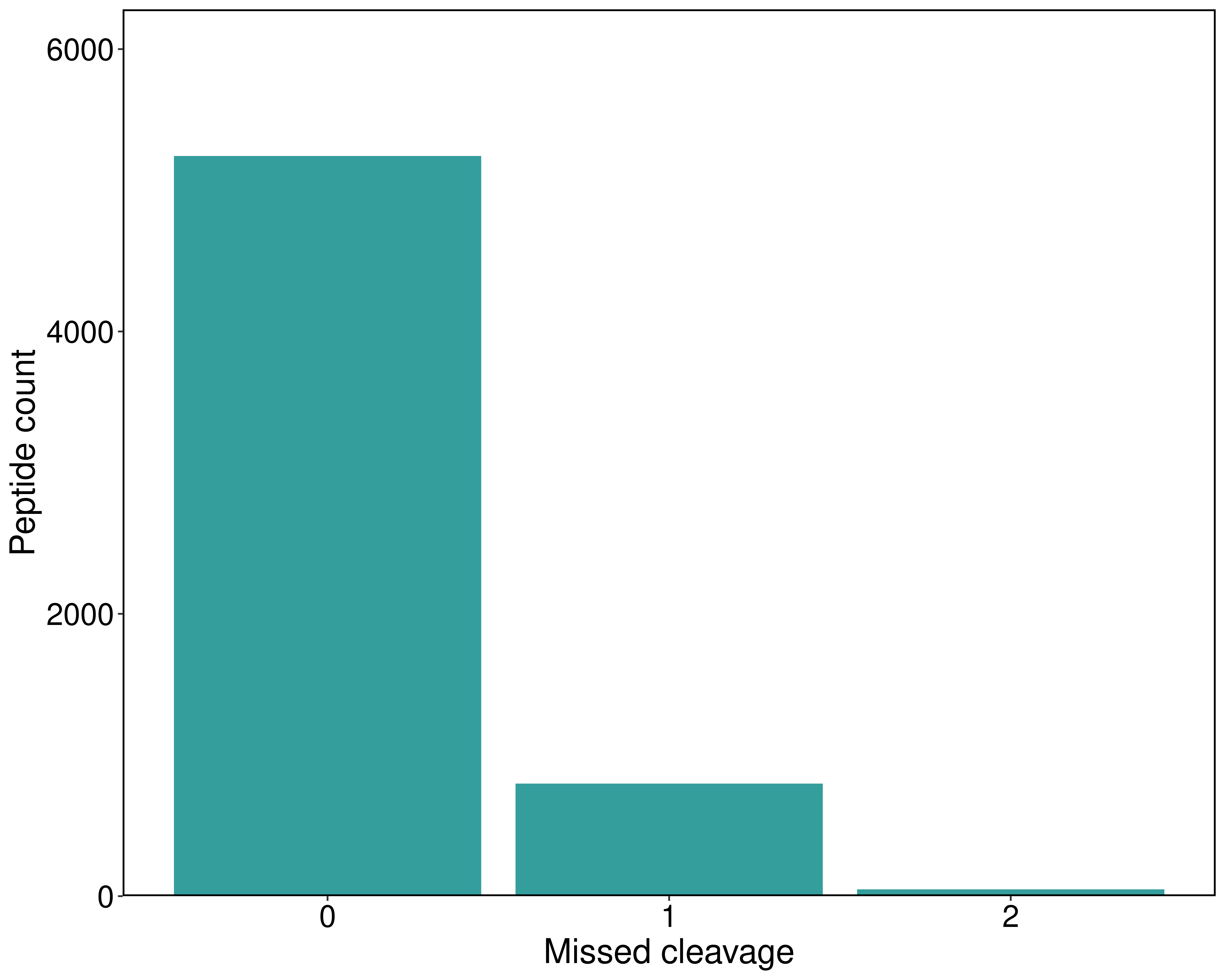

Supplement: Supplementary file 3 [file DataSheet3.zip › Fig. 3 GO and KEGG enrichment analysis of proteins identified by mass spectrometry./supplementary/NC/1.identification/1.3 missing_cleavage.bar.png]

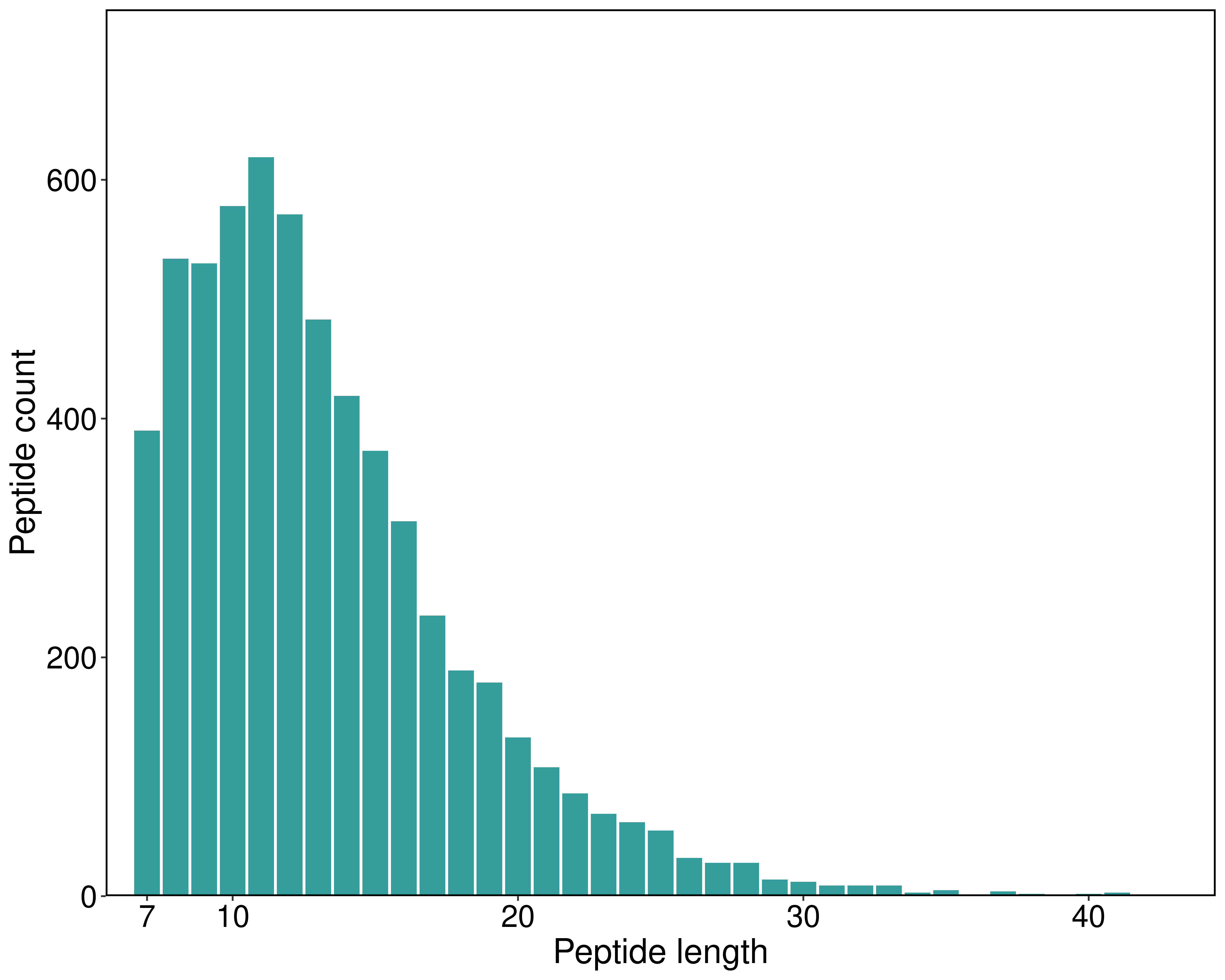

Supplement: Supplementary file 3 [file DataSheet3.zip › Fig. 3 GO and KEGG enrichment analysis of proteins identified by mass spectrometry./supplementary/NC/1.identification/1.1 peptide_length.bar.png]

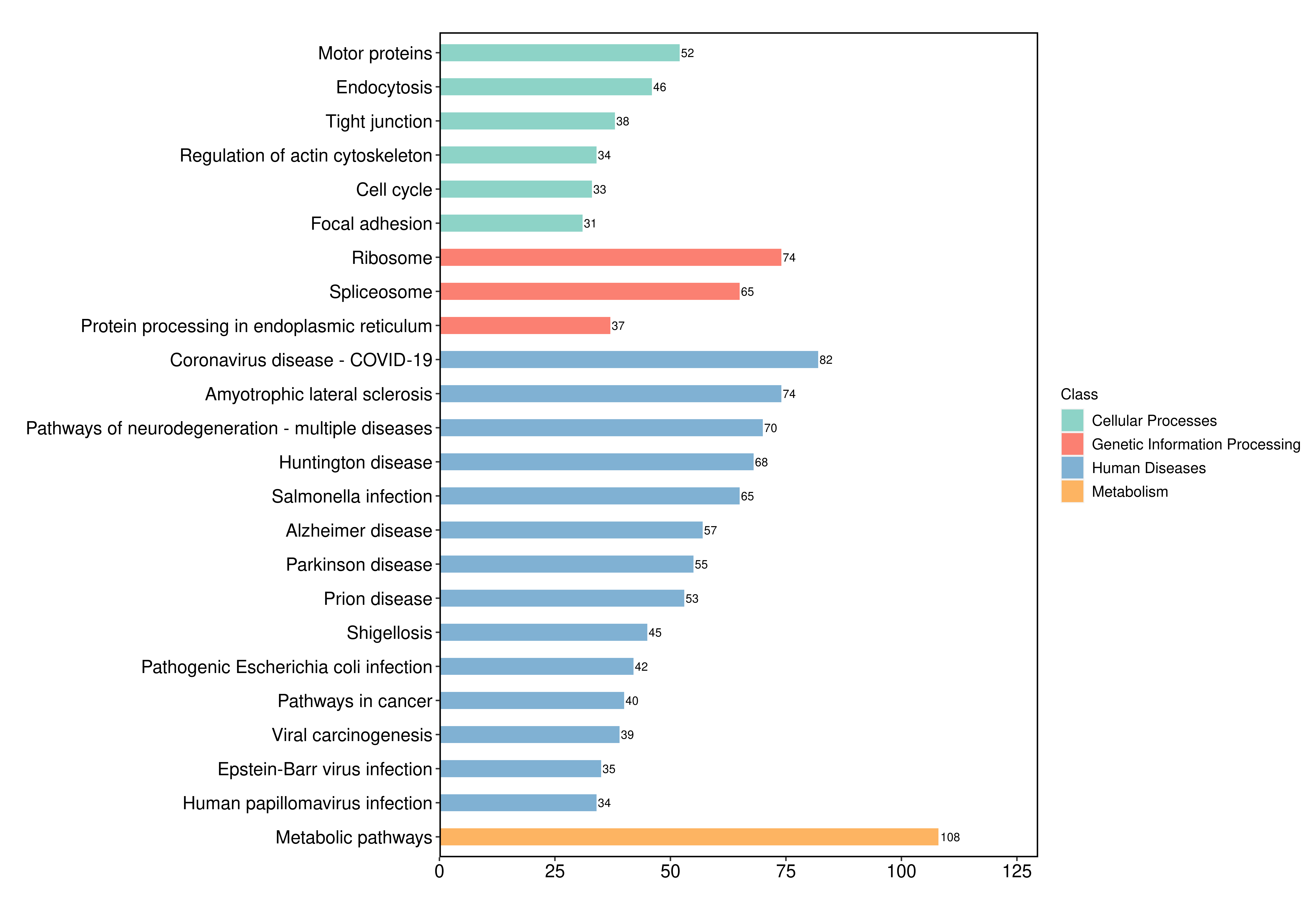

Supplement: Supplementary file 3 [file DataSheet3.zip › Fig. 3 GO and KEGG enrichment analysis of proteins identified by mass spectrometry./supplementary/NC/2.annotation/2.2 KEGG_annotation.bar.png]

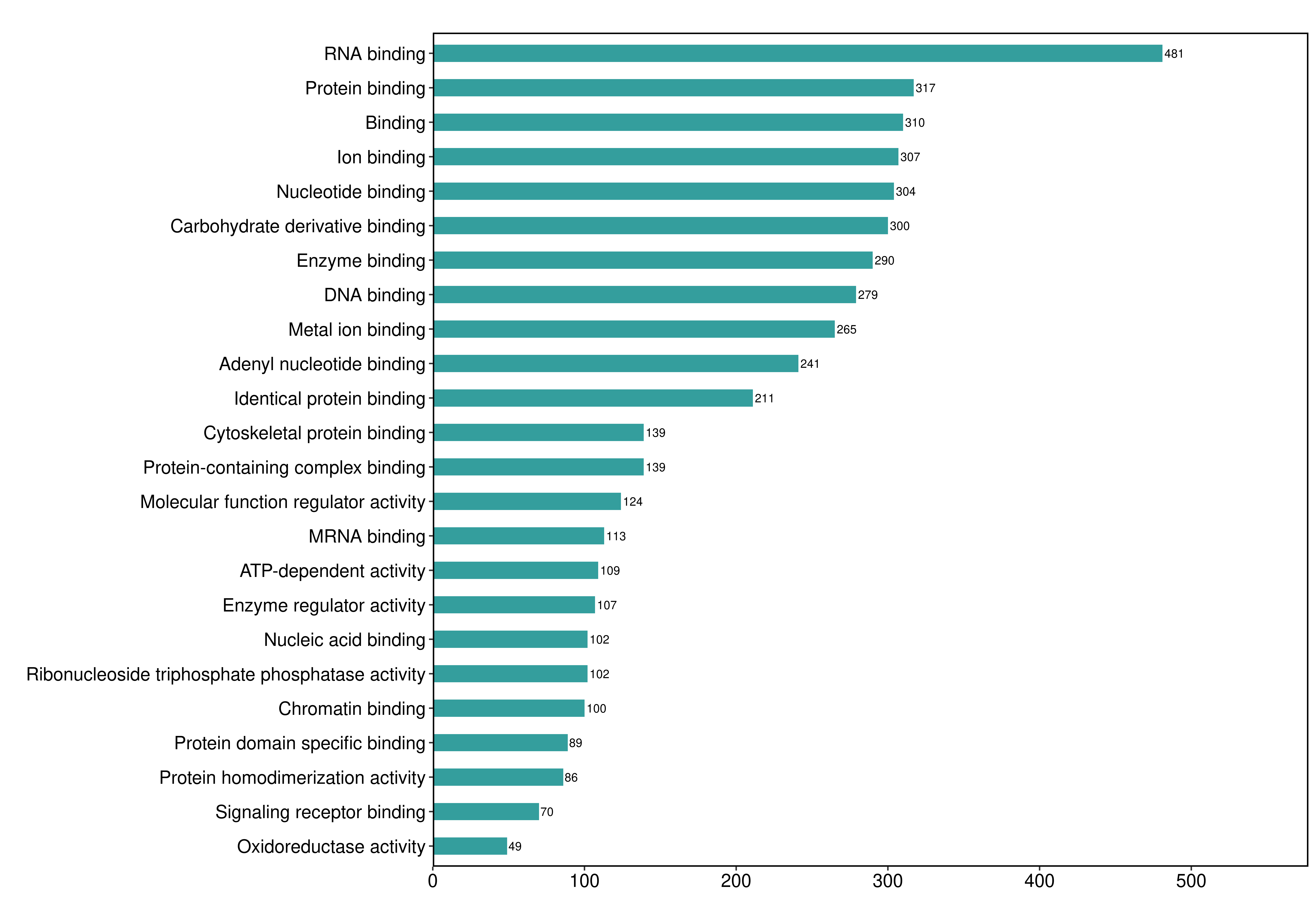

Supplement: Supplementary file 3 [file DataSheet3.zip › Fig. 3 GO and KEGG enrichment analysis of proteins identified by mass spectrometry./supplementary/NC/2.annotation/2.1-3 GOMF_annotation.bar.png]

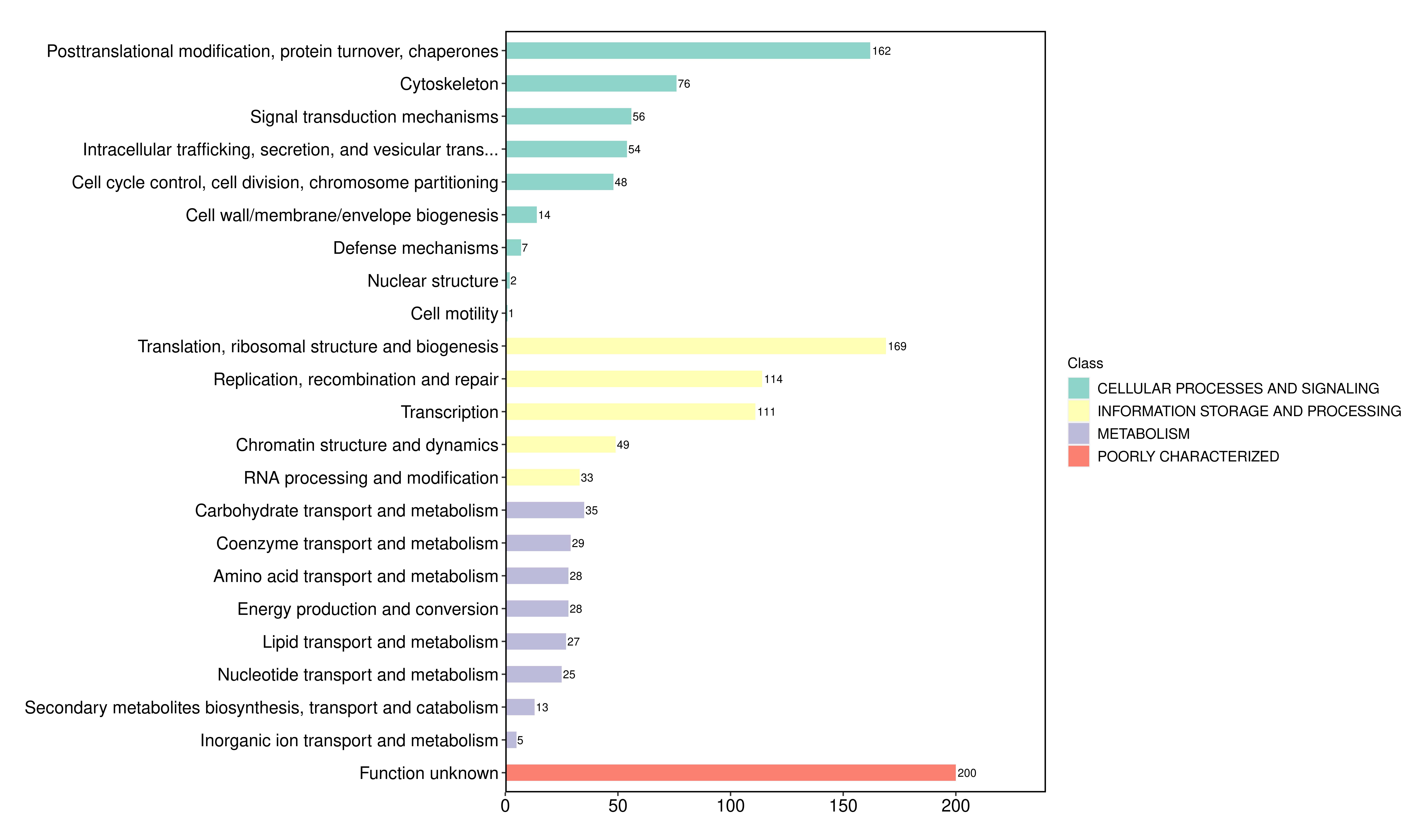

Supplement: Supplementary file 3 [file DataSheet3.zip › Fig. 3 GO and KEGG enrichment analysis of proteins identified by mass spectrometry./supplementary/NC/2.annotation/2.3 COG_annotation.bar.png]

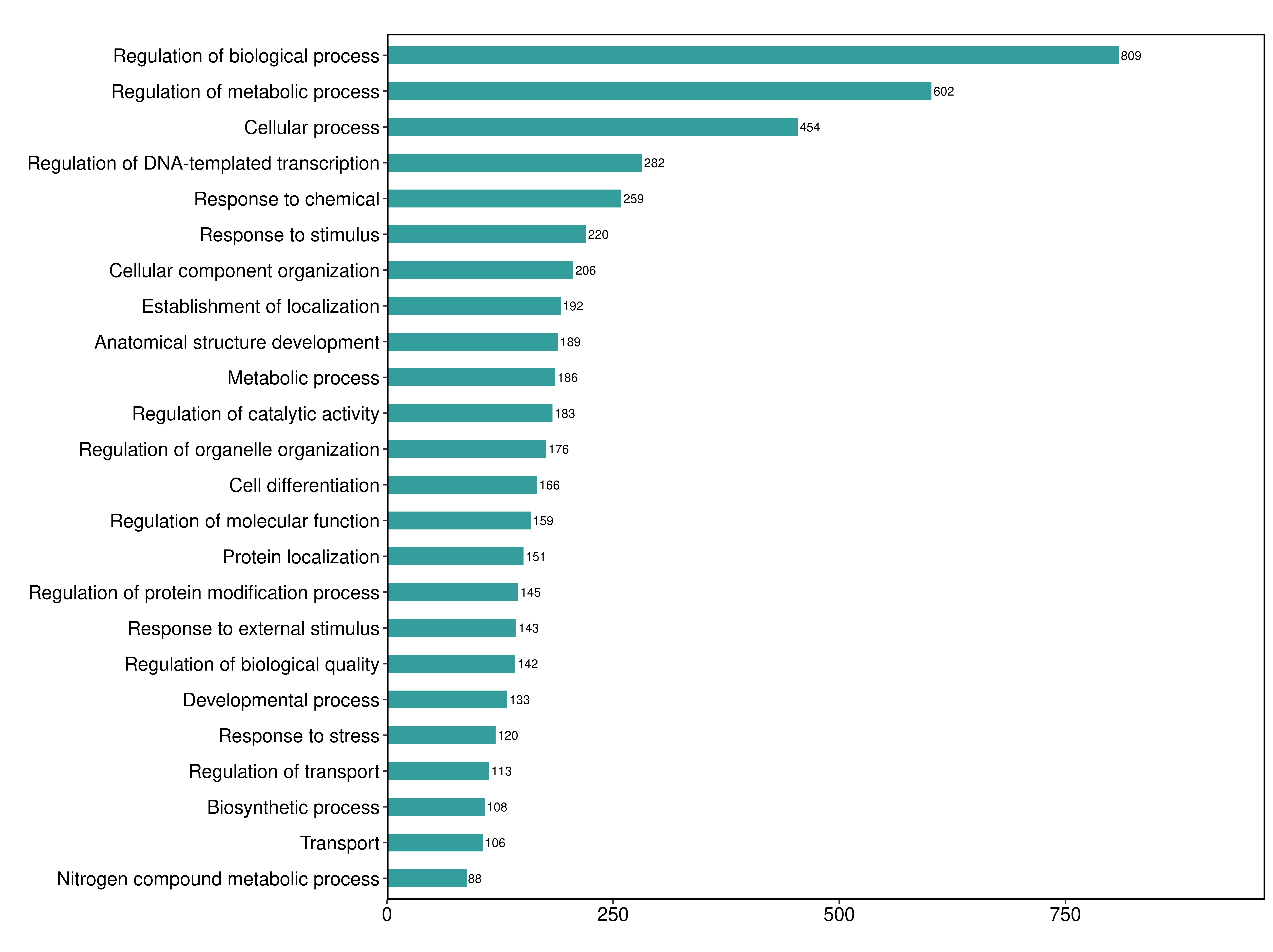

Supplement: Supplementary file 3 [file DataSheet3.zip › Fig. 3 GO and KEGG enrichment analysis of proteins identified by mass spectrometry./supplementary/NC/2.annotation/2.1-1 GOBP_annotation.bar.png]

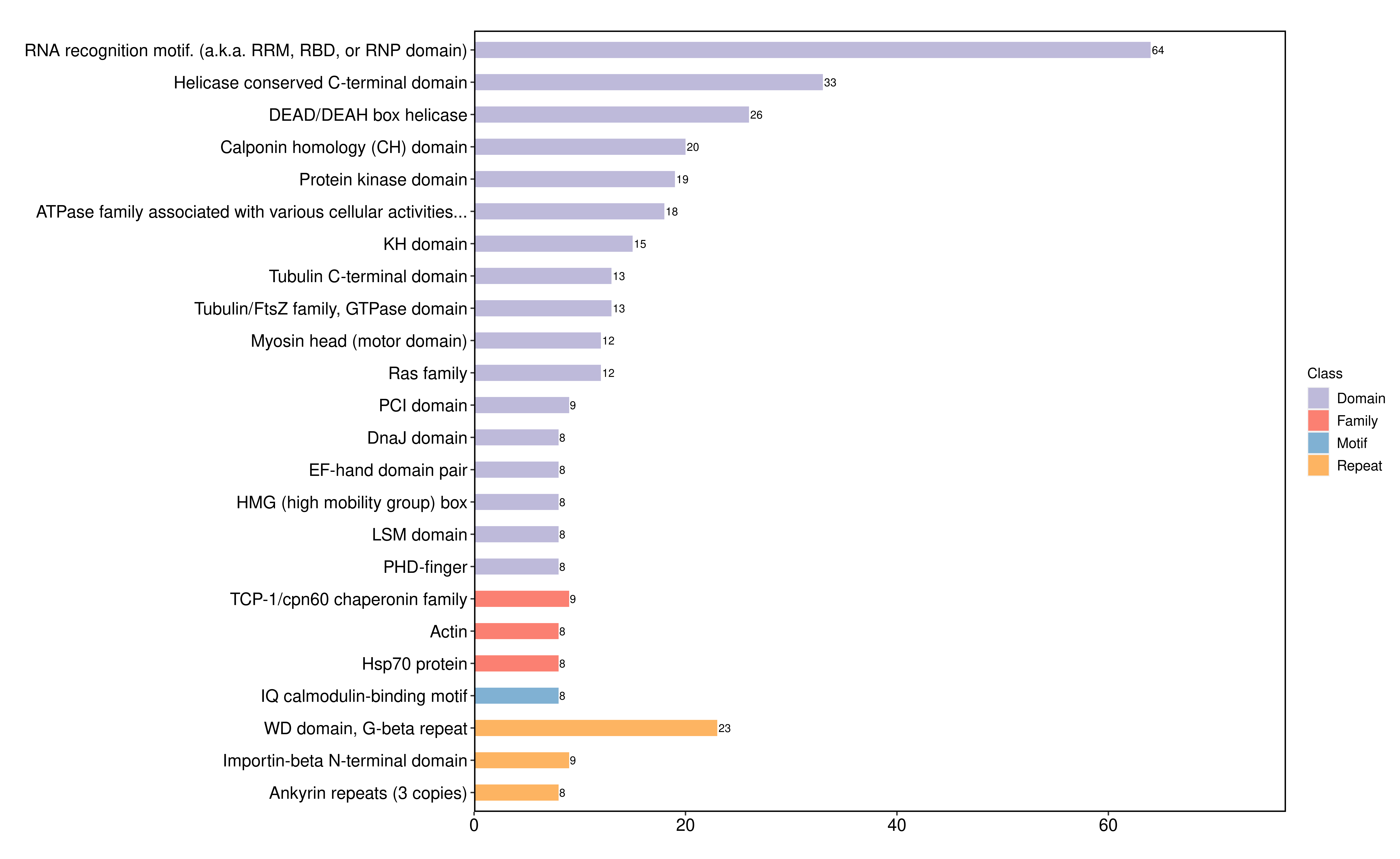

Supplement: Supplementary file 3 [file DataSheet3.zip › Fig. 3 GO and KEGG enrichment analysis of proteins identified by mass spectrometry./supplementary/NC/2.annotation/2.4 Pfam_annotation.bar.png]

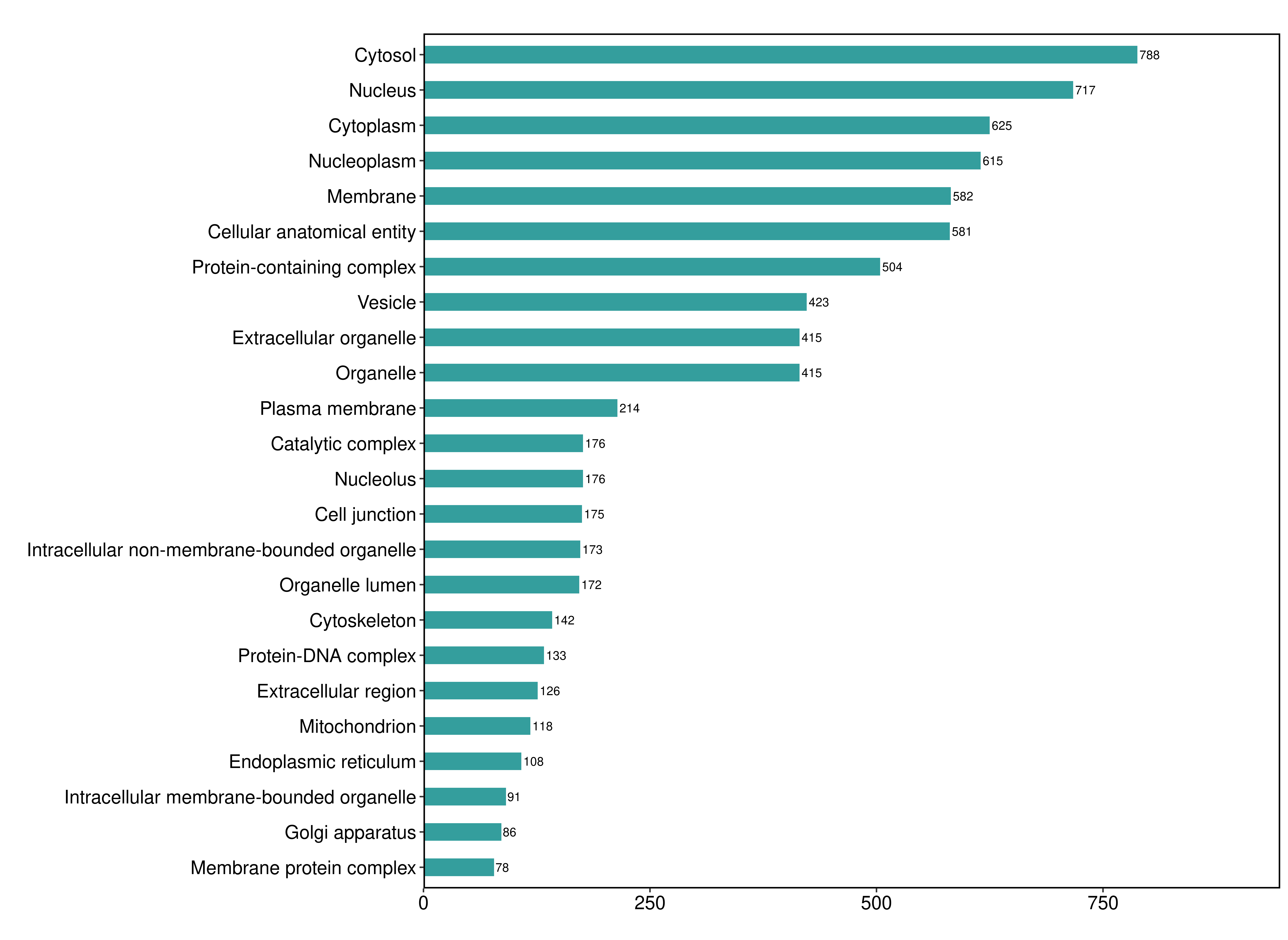

Supplement: Supplementary file 3 [file DataSheet3.zip › Fig. 3 GO and KEGG enrichment analysis of proteins identified by mass spectrometry./supplementary/NC/2.annotation/2.1-2 GOCC_annotation.bar.png]

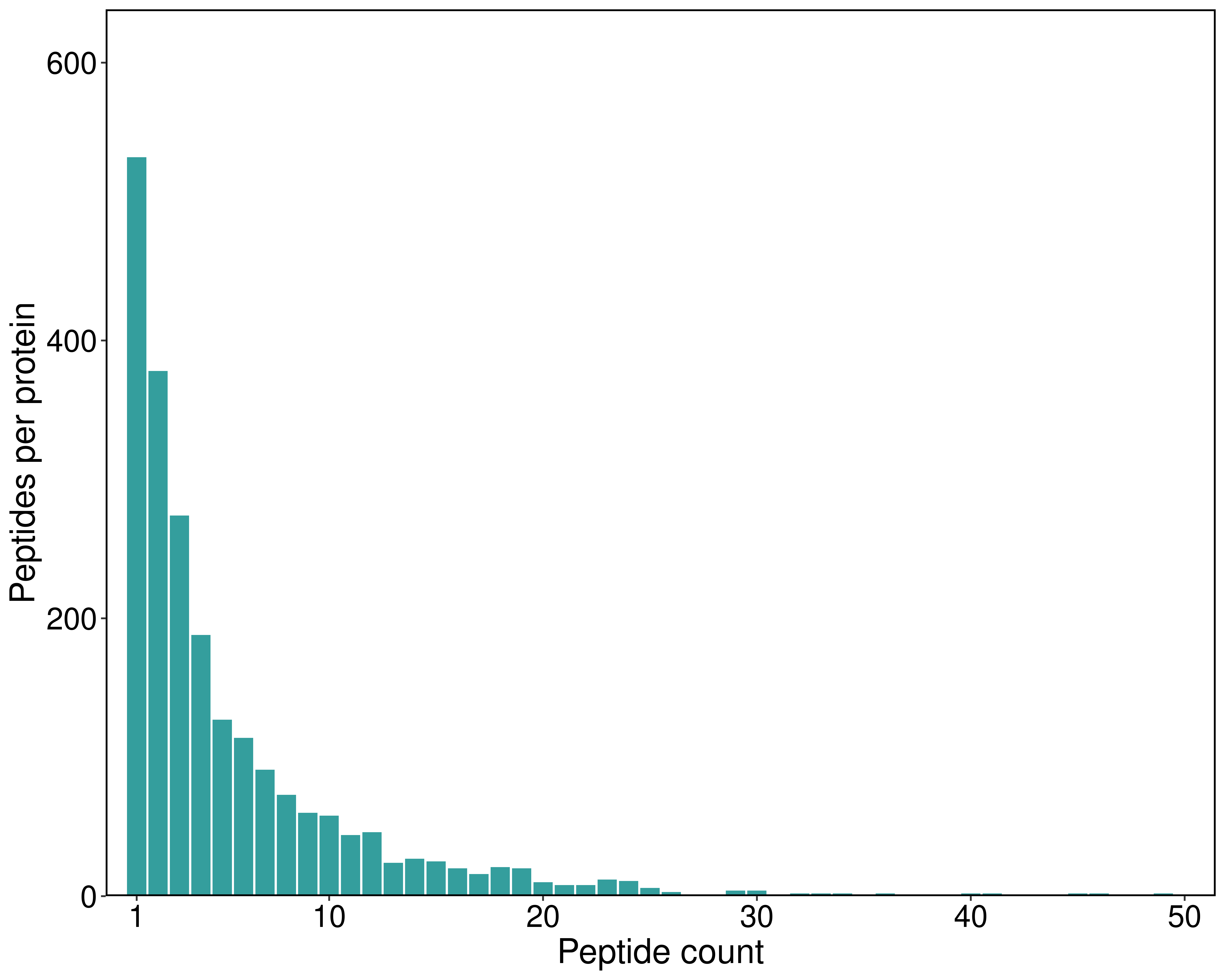

Supplement: Supplementary file 3 [file DataSheet3.zip › Fig. 3 GO and KEGG enrichment analysis of proteins identified by mass spectrometry./supplementary/Sepsis/1.identification/1.2 protein_peptide.bar.png]

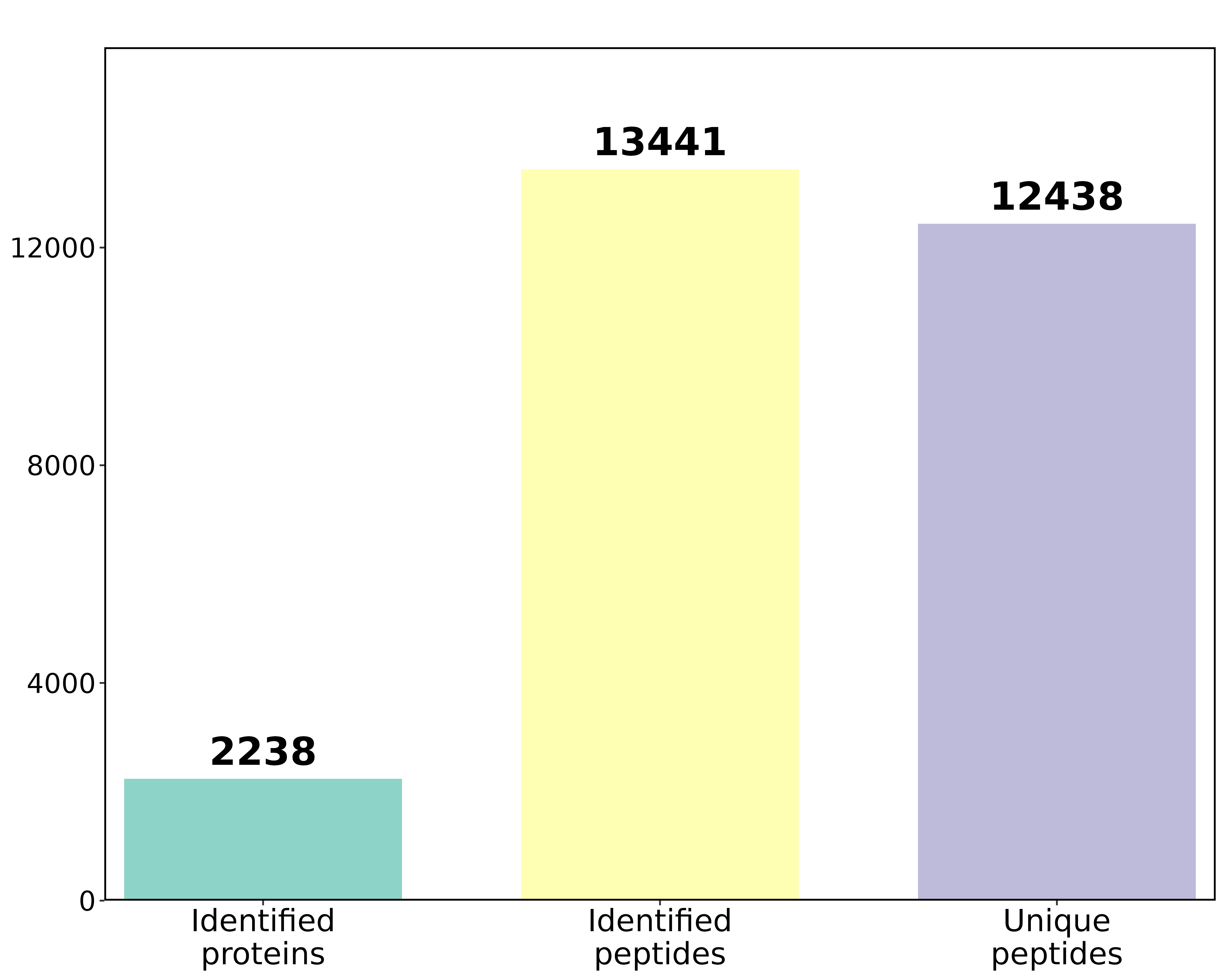

Supplement: Supplementary file 3 [file DataSheet3.zip › Fig. 3 GO and KEGG enrichment analysis of proteins identified by mass spectrometry./supplementary/Sepsis/1.identification/1.4 proteins_stat.bar.png]

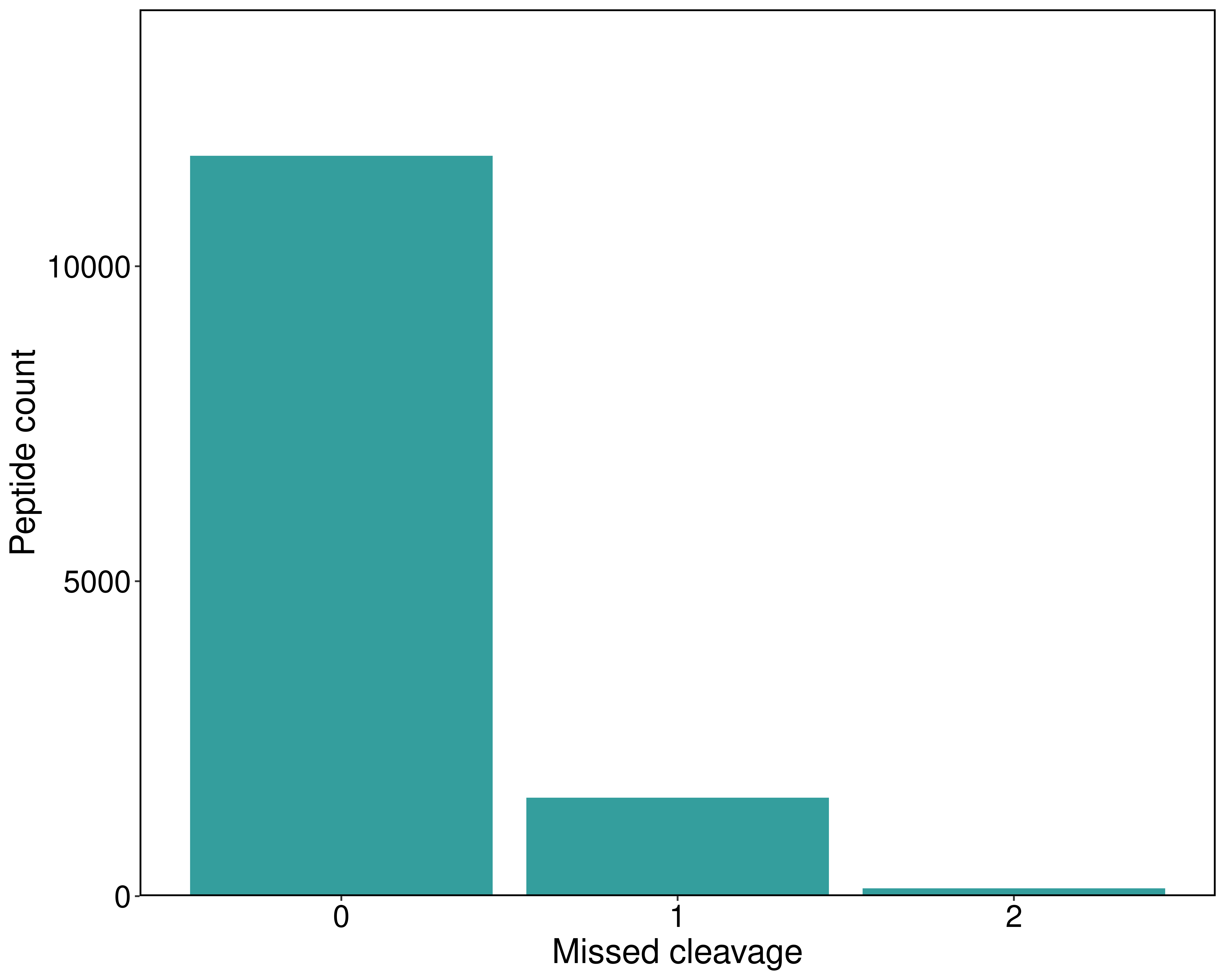

Supplement: Supplementary file 3 [file DataSheet3.zip › Fig. 3 GO and KEGG enrichment analysis of proteins identified by mass spectrometry./supplementary/Sepsis/1.identification/1.3 missing_cleavage.bar.png]

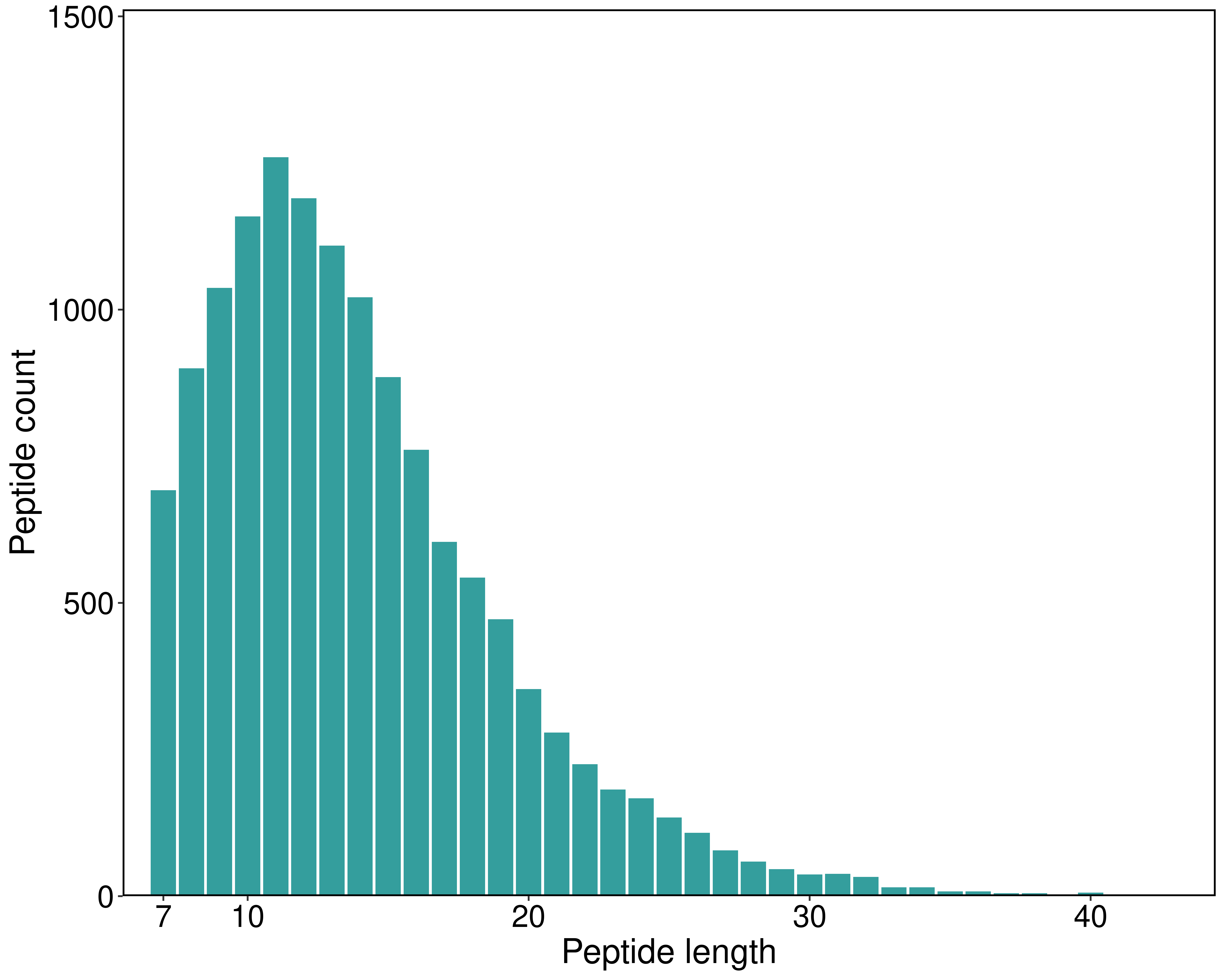

Supplement: Supplementary file 3 [file DataSheet3.zip › Fig. 3 GO and KEGG enrichment analysis of proteins identified by mass spectrometry./supplementary/Sepsis/1.identification/1.1 peptide_length.bar.png]

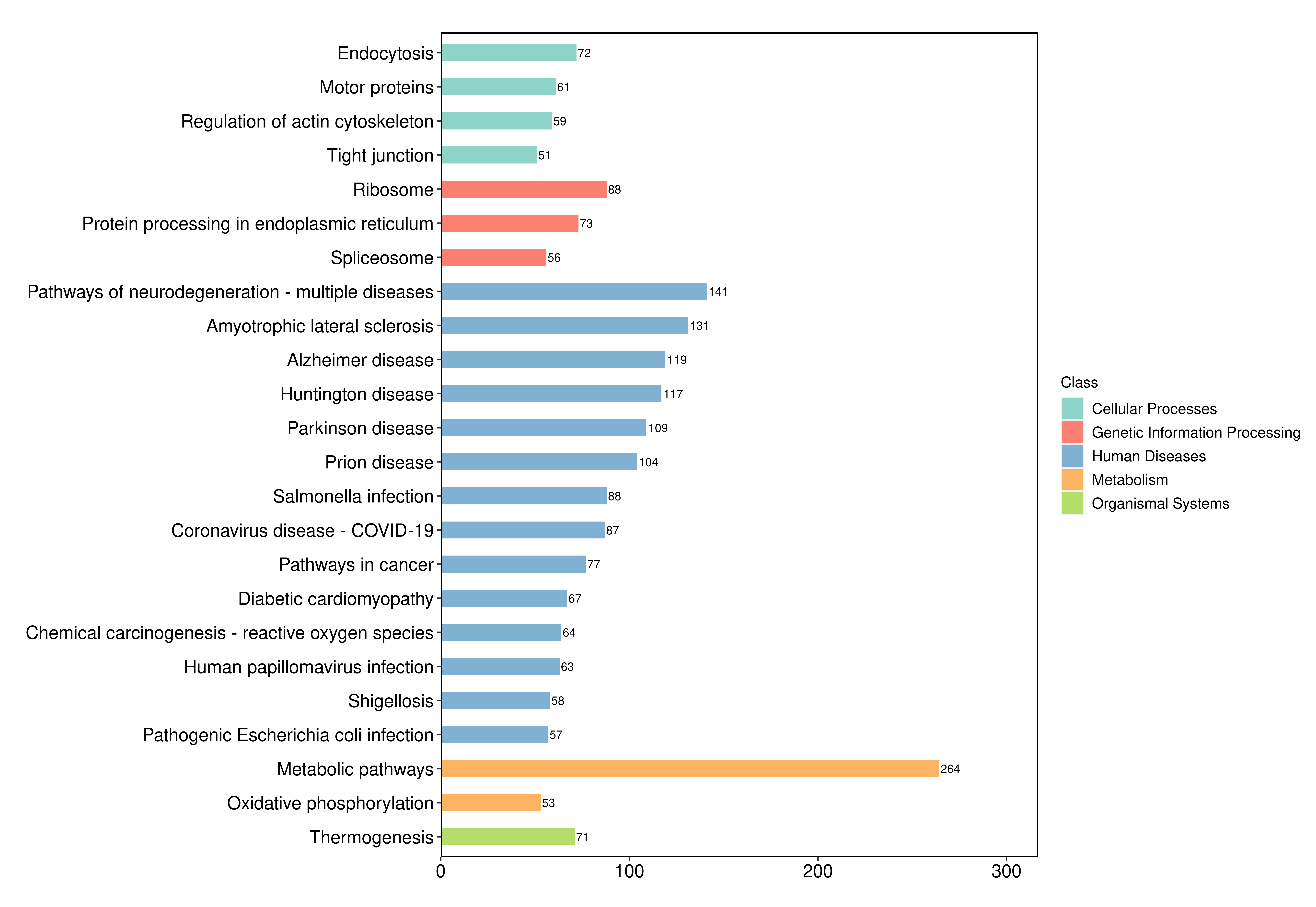

Supplement: Supplementary file 3 [file DataSheet3.zip › Fig. 3 GO and KEGG enrichment analysis of proteins identified by mass spectrometry./supplementary/Sepsis/2.annotation/2.2 KEGG_annotation.bar.png]

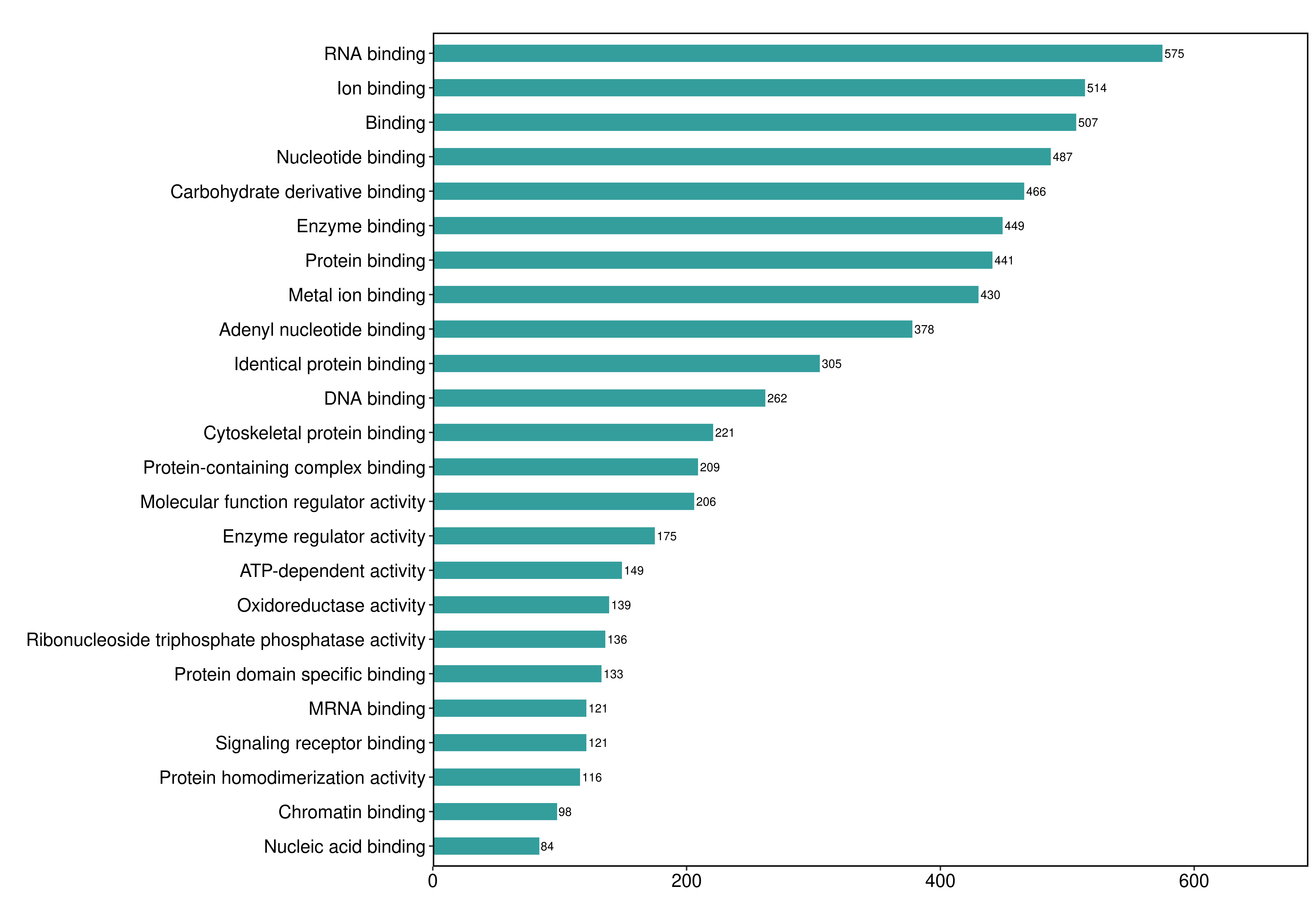

Supplement: Supplementary file 3 [file DataSheet3.zip › Fig. 3 GO and KEGG enrichment analysis of proteins identified by mass spectrometry./supplementary/Sepsis/2.annotation/2.1-3 GOMF_annotation.bar.png]

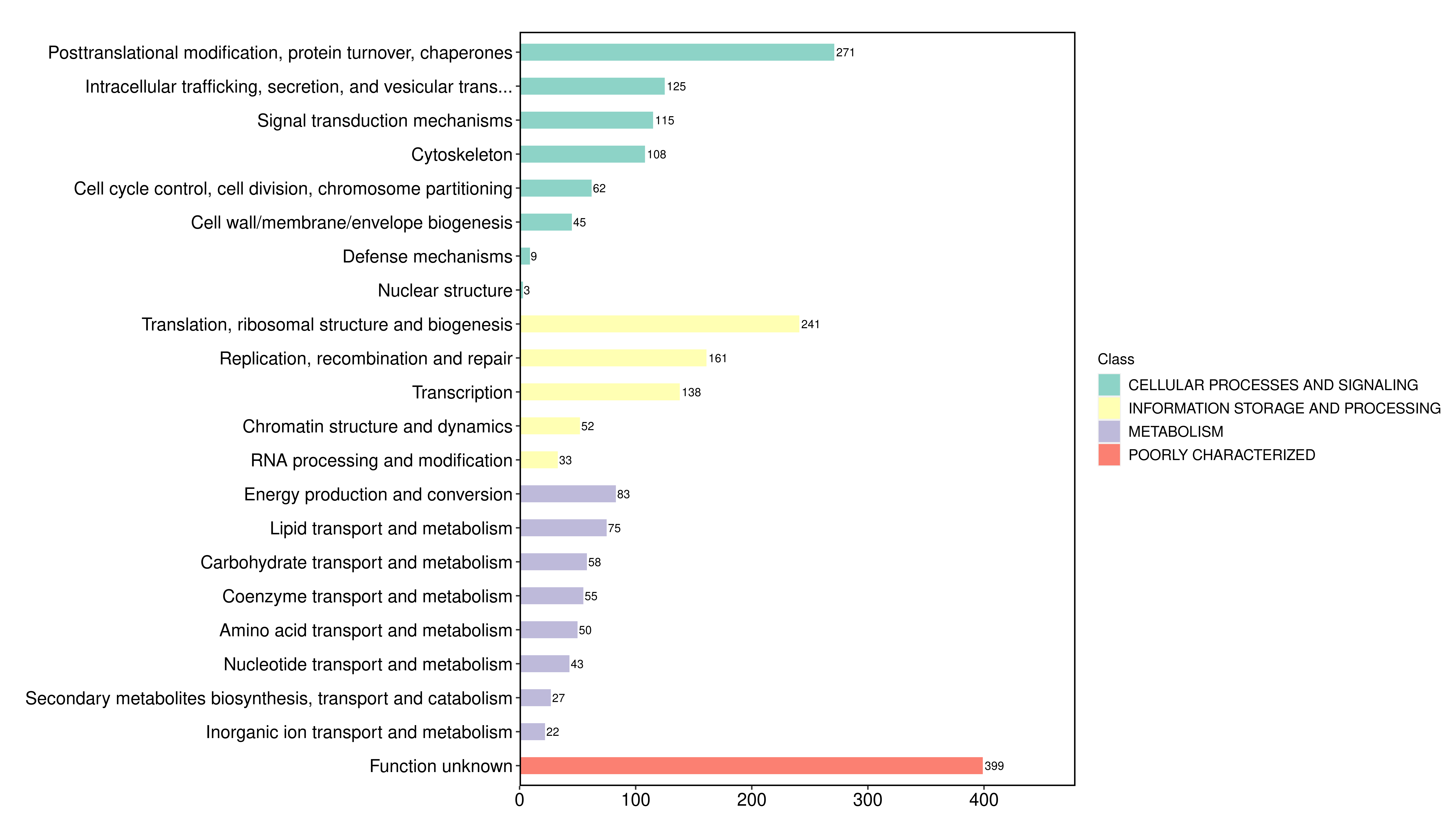

Supplement: Supplementary file 3 [file DataSheet3.zip › Fig. 3 GO and KEGG enrichment analysis of proteins identified by mass spectrometry./supplementary/Sepsis/2.annotation/2.3 COG_annotation.bar.png]

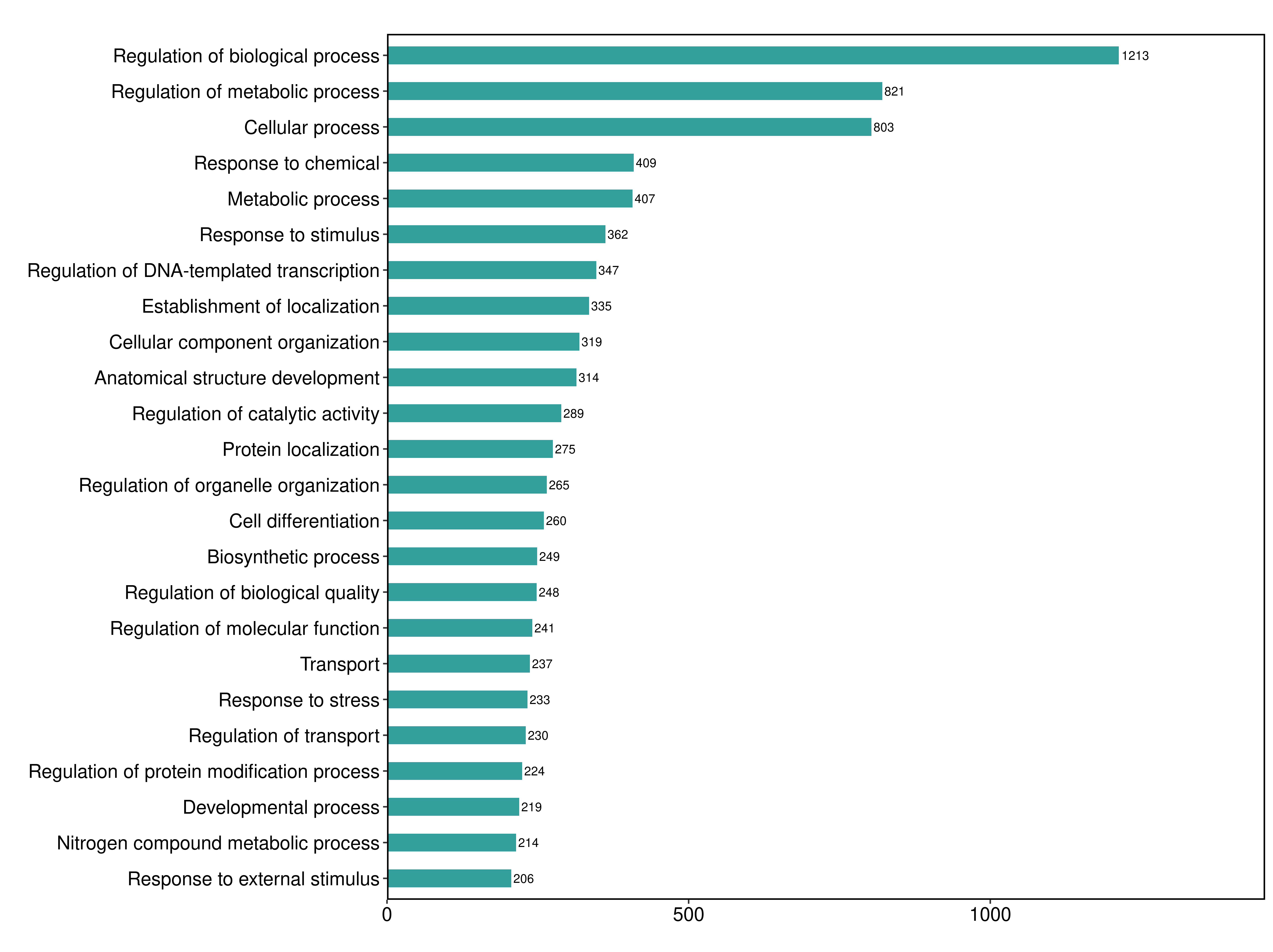

Supplement: Supplementary file 3 [file DataSheet3.zip › Fig. 3 GO and KEGG enrichment analysis of proteins identified by mass spectrometry./supplementary/Sepsis/2.annotation/2.1-1 GOBP_annotation.bar.png]

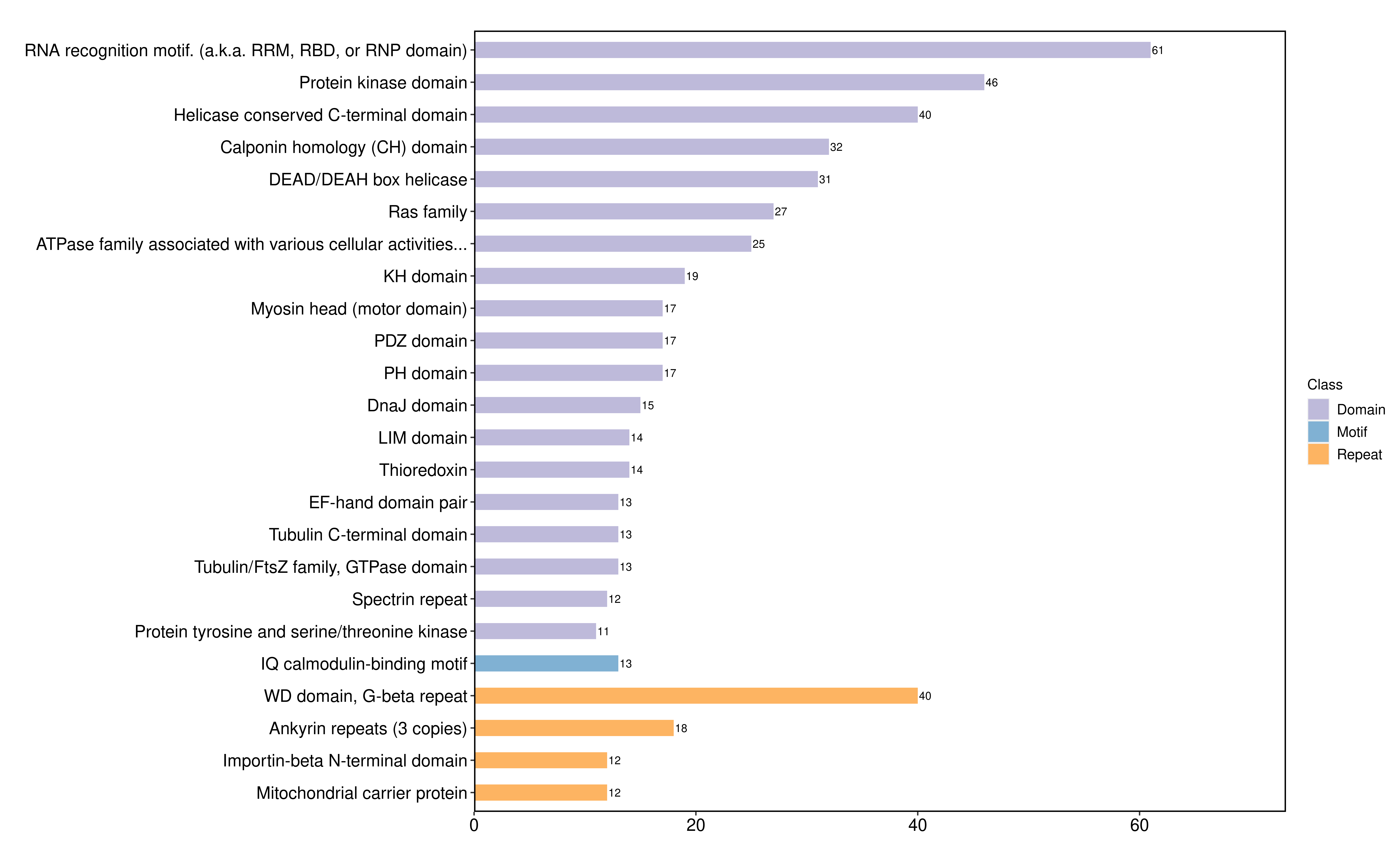

Supplement: Supplementary file 3 [file DataSheet3.zip › Fig. 3 GO and KEGG enrichment analysis of proteins identified by mass spectrometry./supplementary/Sepsis/2.annotation/2.4 Pfam_annotation.bar.png]

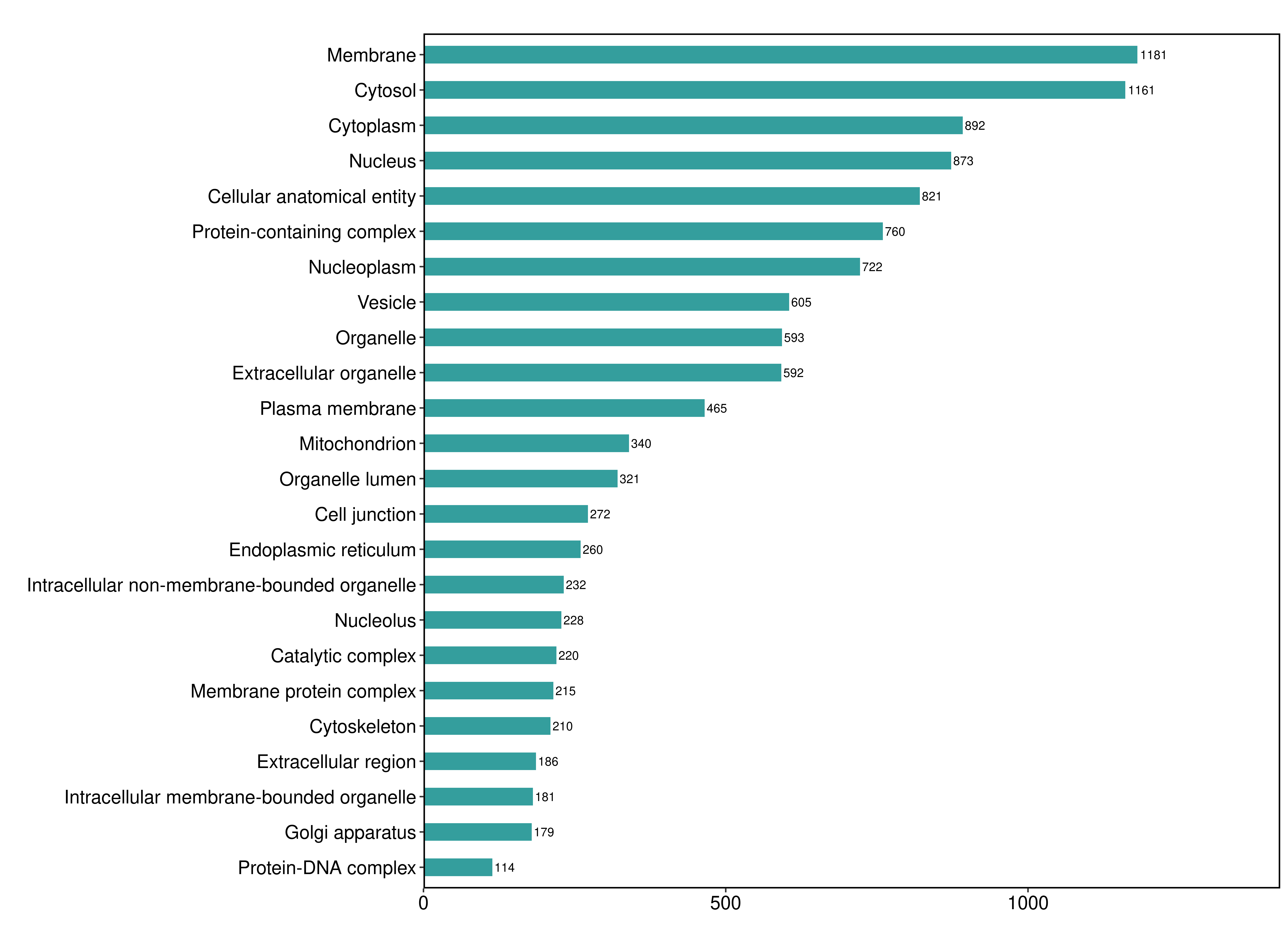

Supplement: Supplementary file 3 [file DataSheet3.zip › Fig. 3 GO and KEGG enrichment analysis of proteins identified by mass spectrometry./supplementary/Sepsis/2.annotation/2.1-2 GOCC_annotation.bar.png]

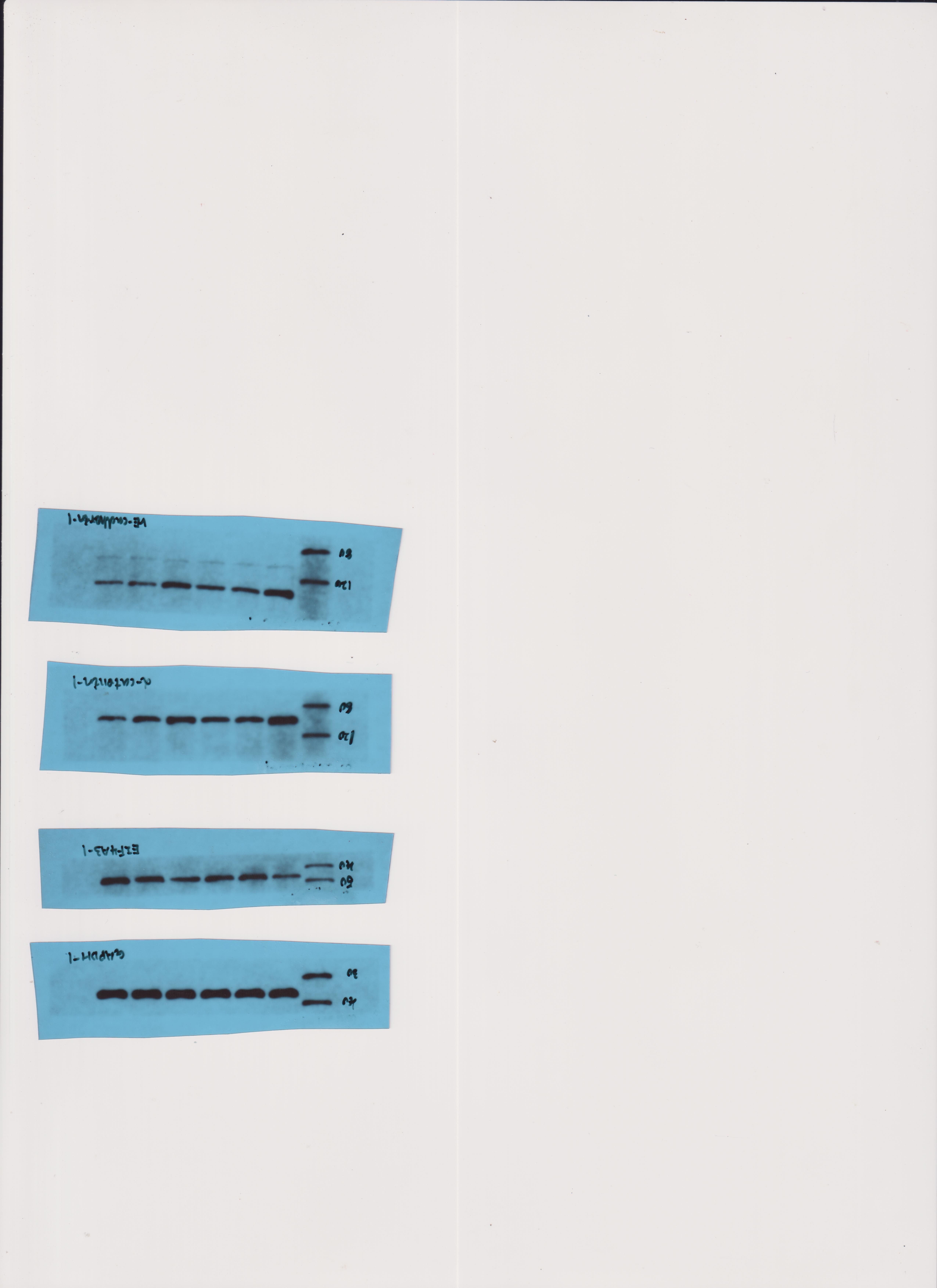

Supplement: Supplementary file 4 [file DataSheet4.zip › Fig.4CπÇüJ∩╝¢5D Western blotting of cellular experiments.jpg]

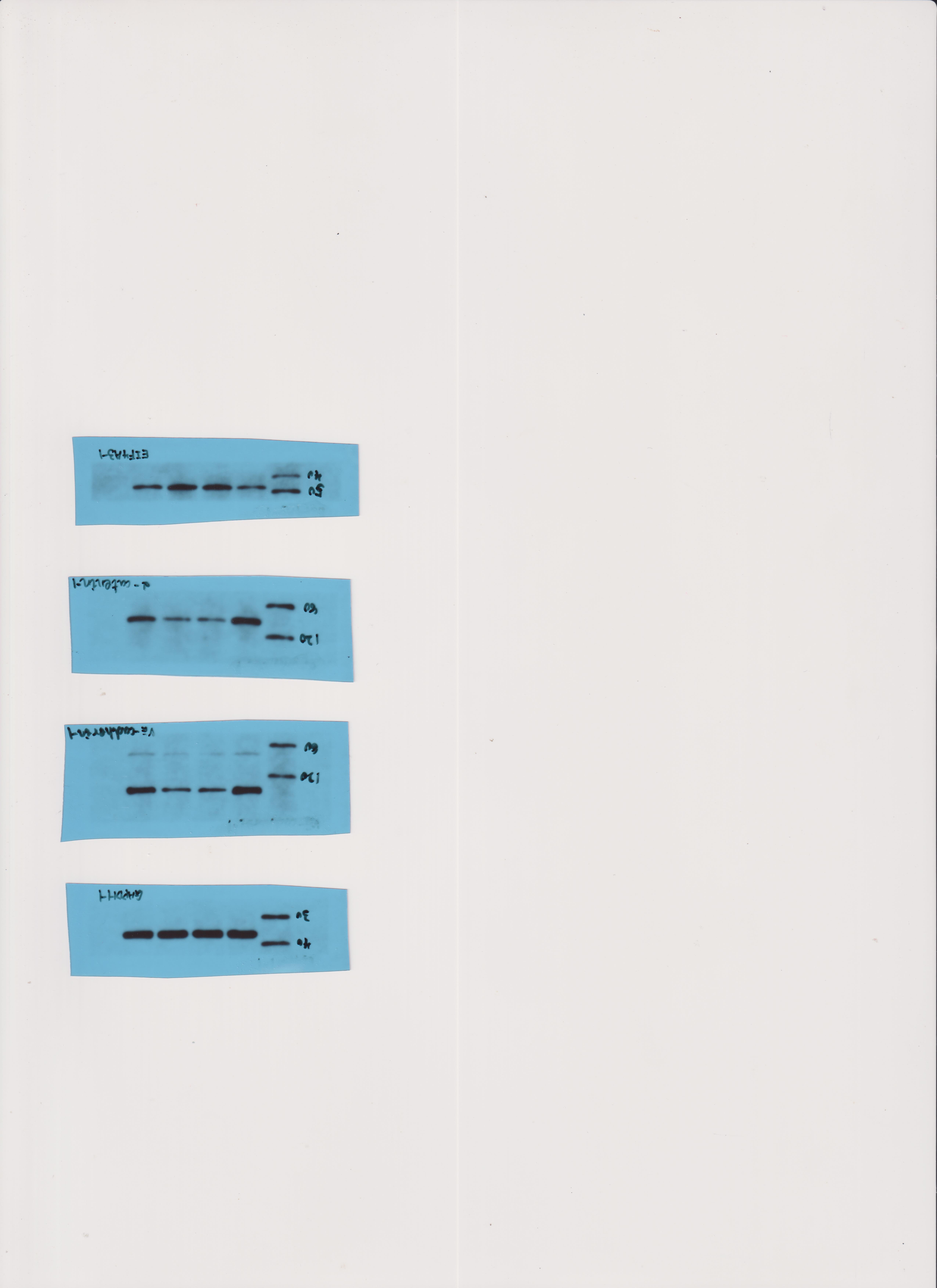

Supplement: Supplementary file 4 [file DataSheet4.zip › Fig.4DπÇüK∩╝¢5C Western blotting of animal experiments .jpg]

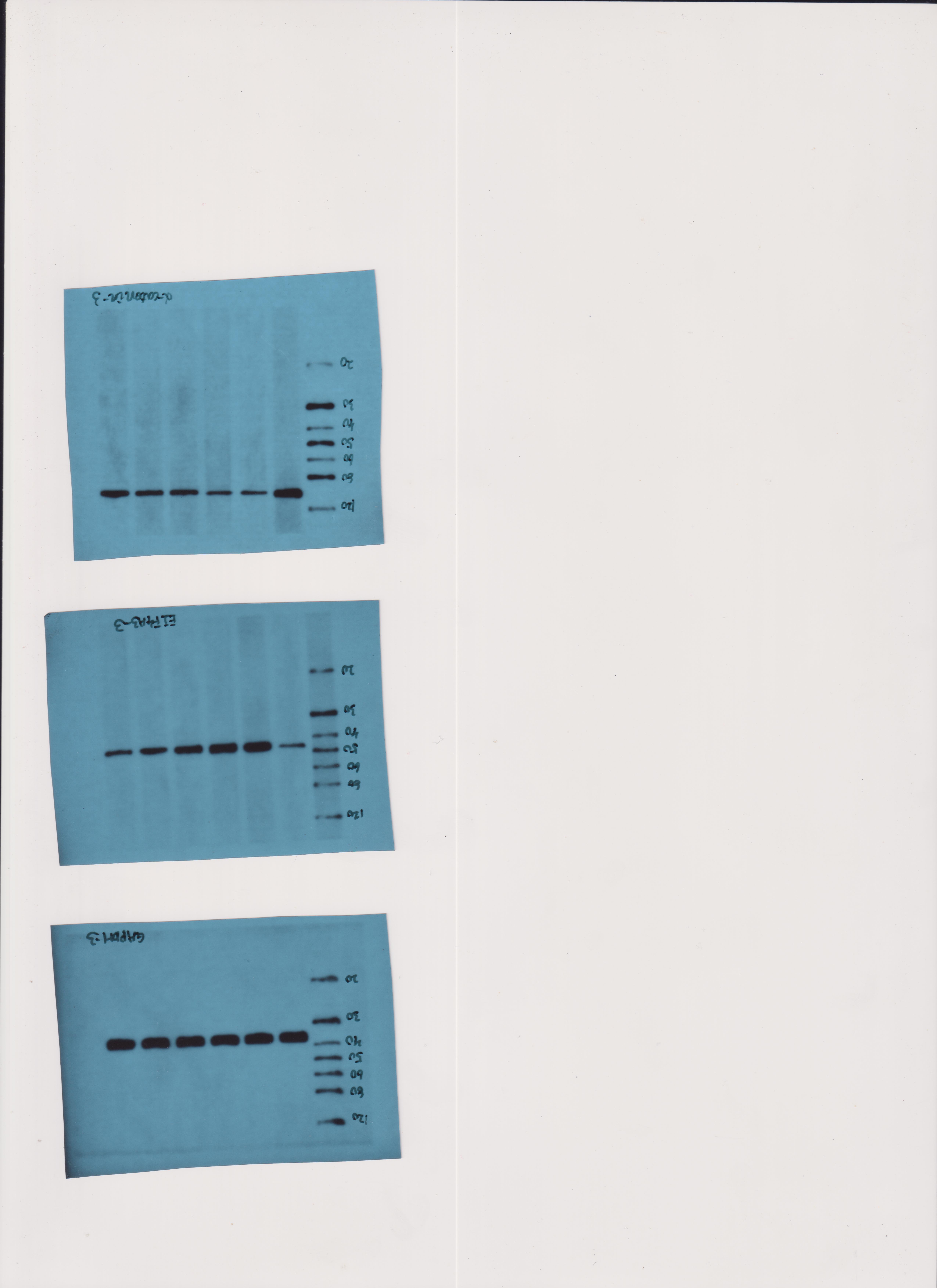

Supplement: Supplementary file 4 [file DataSheet4.zip › Fig.5F Western blotting of cellular experiments.jpg]

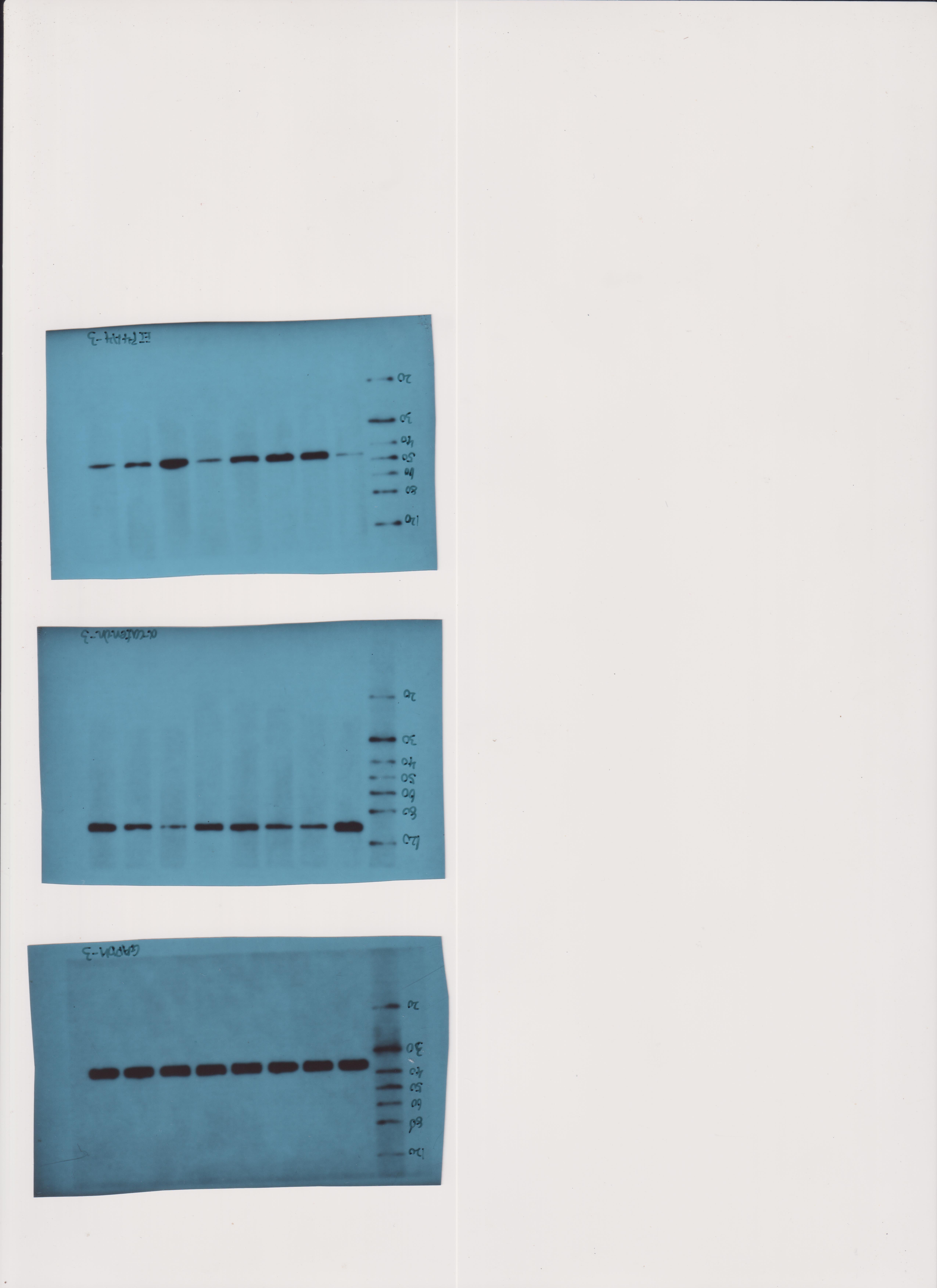

Supplement: Supplementary file 5 [file DataSheet5.zip › Fig.6B Rescue experiment .jpg]

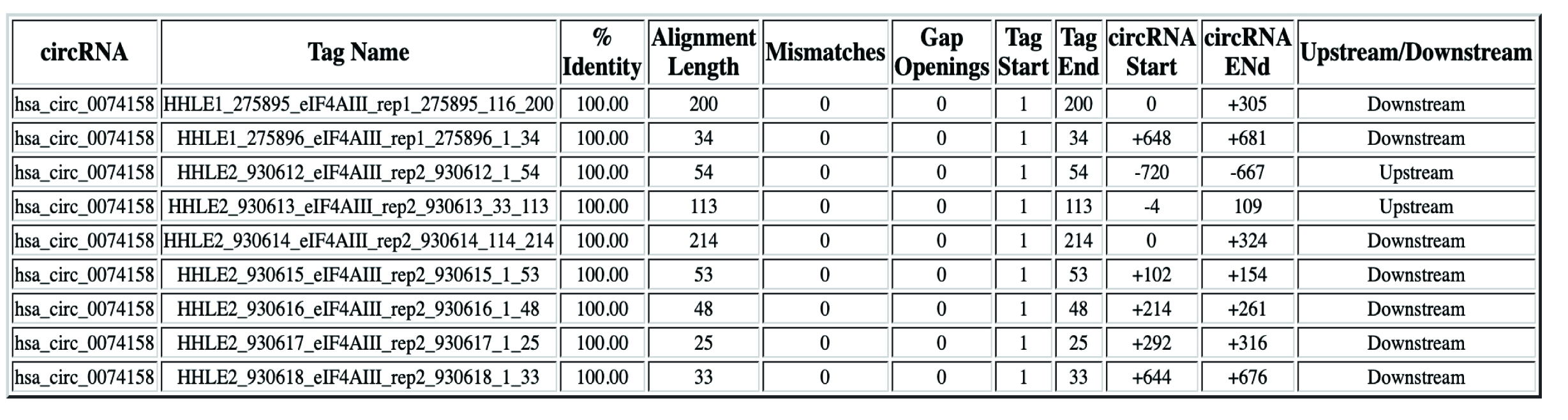

Supplement: Supplementary file 8 [file Image1.tif]
